# Impaired lipid metabolism in astrocytes underlies degeneration of cortical projection neurons in hereditary spastic paraplegia

**DOI:** 10.1186/s40478-020-01088-0

**Published:** 2020-12-07

**Authors:** Yongchao Mou, Yi Dong, Zhenyu Chen, Kyle R. Denton, Michael O. Duff, Craig Blackstone, Su-Chun Zhang, Xue-Jun Li

**Affiliations:** 1 Department of Biomedical Sciences, University of Illinois College of Medicine Rockford, Rockford, IL 61107 USA; 2 Department of Bioengineering, University of Illinois at Chicago, Chicago, IL 60607 USA; 3 Waisman Center, Department of Neuroscience, Department of Neurology, University of Wisconsin, Madison, WI 53705 USA; 4 Department of Neuroscience, University of Connecticut Health Center, Farmington, CT 06030 USA; 5 Department of Genetics and Genome Sciences, University of Connecticut Health Center, Farmington, CT 06030 USA; 6 Cell Biology Section, Neurogenetics Branch, National Institute of Neurological Disorders and Stroke, National Institutes of Health, Bethesda, MD 20892 USA

**Keywords:** Hereditary spastic paraplegia, Human pluripotent stem cells, Cortical projection neurons, Cholesterol homeostasis, Axonal degeneration, Astrocytes

## Abstract

Hereditary spastic paraplegias (HSPs) are caused by a length-dependent axonopathy of long corticospinal neurons, but how axons of these cortical projection neurons (PNs) degenerate remains elusive. We generated isogenic human pluripotent stem cell (hPSC) lines for two *ATL1* missense mutations associated with SPG3A, the most common early-onset autosomal dominant HSP. In hPSC-derived cortical PNs, *ATL1* mutations resulted in reduced axonal outgrowth, impaired axonal transport, and accumulated axonal swellings, recapitulating disease-specific phenotypes. Importantly, *ATL1* mutations dysregulated proteolipid gene expression, reduced lipid droplet size in astrocytes, and unexpectedly disrupted cholesterol transfer from glia to neurons, leading to cholesterol deficiency in SPG3A cortical PNs. Applying cholesterol or conditioned medium from control astrocytes, a major source of cholesterol in the brain, rescued aberrant axonal transport and swellings in SPG3A cortical PNs. Furthermore, treatment with the NR1H2 agonist GW3965 corrected lipid droplet defects in SPG3A astrocytes and promoted cholesterol efflux from astrocytes, leading to restoration of cholesterol levels and rescue of axonal degeneration in SPG3A cortical PNs. These results reveal a non-cell autonomous mechanism underlying axonal degeneration of cortical PNs mediated by impaired cholesterol homeostasis in glia.

## Introduction

Hereditary spastic paraplegias (HSPs) are a large and diverse group of inherited neurodegenerative diseases with the common feature of a length-dependent axonopathy of the corticospinal axons, resulting in spasticity of lower-limb muscles and gait abnormalities [3, 22, 67]. To date, over 80 distinct genetic loci (SPG1-80, plus others) have been associated with HSP [2]. The respective gene products fall into a relatively small number of cellular pathogenic themes, include organelle shaping/distribution, endolysosomal function, intracellular transport, mitochondrial function, myelination, and lipid metabolism [2, 62]. The most common forms—SPG4, SPG3A, and SPG31—are caused by autosomal dominant mutations in proteins that shape and distribute the tubular endoplasmic reticulum (ER) [44, 52]. How mutations in these ER-shaping proteins result in the common axonal degeneration of cortical projection neurons (PNs) remains an enigma.

SPG3A is the most common early-onset, autosomal dominant form of HSP subtype and is caused by mostly missense mutations in the *ATL1* gene that encodes atlastin-1, a membrane-bound, ER-localized dynamin-like GTPase [27, 76]. Atlastin-1 is highly enriched in axonal growth cones in the central nervous system (CNS). Mutation or knockdown of atlastin-1 results in the impairment in neurite architecture, elongation and branching [17, 79], confirming the crucial role of atlastin-1 in the development and maintenance of axons. At the level of ER, *ATL1* mutations impair the proper formation of tubules, vesicles and polygonal networks [27, 38, 46, 52] by inhibiting the formation of three-way ER tubule junctions [50, 75]. These findings suggest that impaired ER structure and ultimately function are associated with an axonopathy of cortical PNs. Recently, several HSP proteins, including atlastin-1 (SPG3A), spastin (SPG4), seipin (SPG17), spartin (SPG20), and REEP1 (SPG31)—which together are mutated in over 50% of HSP patients –regulate the size and/or number of lipid droplets (LDs) in HEK293, COS7, and HeLa cells in vitro, as well as in fat bodies of worms and flies in vivo [14, 15, 19, 30, 51, 65]. Lipid synthesis and metabolism primarily occurs in ER [24]. Hence, aberrant ER structures seen in atlastin-1 mutant cells may be associated with impaired lipids. However, it remains unknown if LDs and lipid metabolism are altered in SPG3A brain and if lipid abnormalities underlie the axonal phenotypes of cortical PNs in HSP.

In vivo studies of SPG3A have emphasized the use of *Drosophila* and zebrafish models, which exhibit aberrant nerve outgrowth and locomotion deficits caused by defects in spinal motor neuron axons [17, 34]. There are currently no rodent models of SPG3A that allow examination of cortical PNs, the cell type affected in HSP. By establishing isogenic human pluripotent stem cell (hPSC) lines with two different *ATL1* missense mutations, we now show disease-specific axonal defects in hPSC-derived cortical PNs but not spinal motor neurons, recapitulating a hallmark pathology of SPG3A. Assays of lipids reveal a significantly reduced LD size specifically in SPG3A astrocytes, the cells in the mature brain for synthesizing and storing lipids [13, 56], but not in cortical PNs. SPG3A astrocytes also display reduced cholesterol efflux. Direct application of cholesterol, or simply promoting cholesterol efflux from astrocytes in mixed neuronal and glial cultures, rescues the axonal defects. Thus, we identify a non-cell autonomous mechanism of cortical PN degeneration in SPG3A via impaired lipid dynamics in astrocytes.

## Materials and methods

### Ethics statement

All experiments involving hESCs and iPSCs were approved by the University of Illinois Embryonic Stem Cell Research Oversight Committee (ESCRO) and IBC.

### Human pluripotent stem cell (hPSC) cultures

hESCs (passage 20–40) comprised H9, ATL1-A161P #70, ATL1-A161P #4, and iPSCs (passage 20–40) included GM1 (labeling as WT), SPG3A patient-derived iPSCs (labeling as ATL1-P342S) [77], and ATL1-342-Cor. All hPSCs were maintained on irradiated mouse embryonic fibroblast (irMEF) feeder layers in 10 ng/ml FGF-2 (PeproTech)-supplemented hESC medium containing DMEM/F12 (Gibco), 1 × non-essential amino acids (NEAA, Gibco), 20% Knockout Serum Replacement (Gibco), 0.5 × GlutaMax (Gibco), and 0.1 mM β-mercaptoethanol (Sigma-Aldrich). Cell culture was performed as previously described [4, 77]. ATL1-A161P #70 heterozygous and ATL1-A161P #4 homozygous isogenic cell lines were generated from H9 hESCs using CRISPR-Cas9-mediated homologous recombination. The ATL1-342-Cor iPSC line was genetically corrected from ATL1-P342S iPSCs.

### Cortical PNs differentiation

hPSCs including hESCs (H9, ATL1-A161P #70 and ATL1-A161P #4) and iPSCs (wild-type, ATL1-P342S and ATL1-342-Cor) were used for generation of cortical PNs as previously reported [4, 36, 77]. Briefly, hPSCs were dissociated with 1 mg/ml Dispase (Gibco) for 2 min (min) at 37 °C and cultured in suspension to generate embryonic bodies (EBs) at D0 in hESC medium without FGF2 for 4 days. Medium was replenished every day until D4. Neural induction medium (NIM) was prepared by supplementing DMEM/F12 medium with 1 × N2 (Gemini Bio-Products), 2 µg/ml heparin (Sigma-Aldrich), and 1 × NEAA. NIM medium supplemented with 2 µM DMH1 (Selleckchem) and 2 µM SB431532 (Stemgent) was used for EB culture at D4. At D7, EBs were collected and plated in NIM with 5% fetal bovine serum (FBS, Atlas Biologicals) on six-well plates overnight to attach to the wells; NIM without any supplementation was used to induce EB differentiation into neuroepithelial (NE) cells from D8 to D17. The medium was changed every other day until D17. At D17, NE cells were isolated and cultured in NIM with 1x B27 (Gemini Bio-Products), 1 μM cAMP (Sigma-Aldrich), and 10 ng/ml IGF-1 (PeproTech) in suspension to obtain neurospheres. Half of the old medium was replenished every other day. After D42, neurospheres were plated on 1 mg/ml poly-ornithine (Sigma-Aldrich) and Geltrex (Gibco)-coated coverslips. To generate cortical PNs in regular neural cultures, the neural differentiation medium (NDM) was used with half of the medium changed every other day after the neurospheres were attached on coverslips. In regular neural cultures, the NDM contained Neurobasal medium (Gibco), 1 × N2, 1 × B27, 1 μM cAMP, 10 ng/ml IGF-1, 10 ng/ml hBDNF (PeproTech) and 10 ng/ml hGDNF (PeproTech). The cortical PNs at 6 weeks (D42) were used for examining axonal length. Long-term cultures (around 3 months) were assessed for axonal abnormalities (swellings). Axonal transport, cholesterol content and efflux were examined in cultures during the early stage of degeneration (between 7 and 11 weeks). In the enriched cortical PNs cultures, NDM with 0.1 μM compound E (Calbiochem) was used for neural cultures after neurospheres were plated on coverslips.

### Differentiation of cortical astroglial cells from hPSCs

Cortical astroglial cells were differentiated from hPSCs using previously reported methods [31]. Methods for astroglial cell differentiation from hPSCs between D0 and D21 were the as same as for cortical PNs in the period of D0-D21. On D21, cortical neurospheres were cultured and expanded in NIM supplemented with 10 ng/ml hEGF (Sigma-Aldrich) and 10 ng/ml FGF2. Medium for astroglial cells was replenished every 2–3 days. After 6 months of differentiation, cortical-glial spheres were dissociated with Accutase (Life Technologies) and plated on polyornithine- and Geltrex-coated coverslips. NIM with 10 ng/ml CNTF (R&D Systems) was used for maturation of astroglial cells for 6 days after attachment. Six-month-old astroglial cells were collected for immunostaining, determination of cholesterol efflux, and cholesterol efflux-associated changes in mRNA levels.

### Astroglia-conditioned medium preparation

Astroglia-conditioned medium was prepared as previously published with modifications [71]. Astroglial cells were seeded onto six-well plates (~ 800,000/well) and then exposed to NIM for 24 h. Conditioned medium was collected and centrifuged at 1000×*g* for 4 min and then concentrated fivefold using Amicon Ultra-4 filter devices (10 kD, Millipore). A mixture of the concentrated wild-type and H9 astroglia-conditioned medium with an equal volume of Neurobasal medium supplemented with 1 μM cAMP and 10 ng/ml IGF-1 was used for culturing ATL1-P342S and ATL1-A161P #70 cortical PNs to assess any effects on axonal swellings. A mixture of NIM with an equal volume of Neurobasal medium supplemented with 1 μM cAMP and 10 ng/ml IGF-1 was used as a control.

### Axonal outgrowth and swellings

Axonal outgrowth of hPSC-derived cortical PNs was determined at D42 of differentiation. Neurospheres were incubated with Accutase for 2 min at 37 °C and dissociated into small clusters; trypsin inhibitor was added to the mixture of Accutase and neurospheres. Dissociated cells were plated onto coverslips and immunostained for Ctip2 and Tau. Axons of Ctip2^+^ cortical PNs were measured using Fiji software.

Axonal swellings were determined by immunostaining for Tau in long-term cultures (over 3 months). At least five blindly-selected fields on three coverslips for each group were imaged. The number of axonal swellings (defined as a diameter > 2 times that of the diameter of the contiguous axon) was counted and divided by the total axonal length in each field, which was measured using Fiji software [9, 32]. Effects of GW3965 on axonal swellings were determined using the same methods for staining and quantification. 5 µM GW3965 (Sigma-Aldrich) was used to treat ATL1-P342S and ATL1-A161P #70 cortical PNs in regular neural cultures for 7 days. DMSO (Thermo Fisher)-treated neurons were used as controls.

### Cholesterol cell-based detection

A cholesterol cell-based detection assay kit (Cat#: 10009779, Cayman Chemical) was used for measurement of cholesterol in neuronal cell bodies and axons. Neural cells were fixed using the cell-based assay fixative solution for 10 min, then incubated with Filipin III solution for 60 min and examined using an Olympus IX83 microscope with the same exposure time. At least five images were randomly taken from each of three replicate coverslips. The Filipin average intensities were quantified using Fiji software as previously described [60]. Average Filipin intensities in neuronal cell bodies and axons were traced using “segmented line” tool in Fiji software, and the “profile plot” function in Fiji software was used to obtain the pixel intensity of Filipin in neuronal cell bodies and axons. Axons were identified based on morphological criteria (constant thin diameter, long neurites with no branching, and direct emergence from the cell body), as described previously [77]. For determining effects of GW3965 on cholesterol content, ATL1-P342S and ATL1-A161P #70 cortical PNs in regular neural cultures were treated with 5 µM GW3965 for 3 days following the same procedures for Filipin staining and quantification. DMSO-treated neurons were used as control. Double staining of Filipin with the axonal marker Tau was performed as previously described [6]. Briefly, neurons were fixed with ice-cold 4% paraformaldehyde (Sigma-Aldrich) in PBS for 20 min. Neurons were then incubated in the Filipin solution supplemented with 10% fetal bovine serum for 2 h at room temperature, followed by primary rabbit anti-Tau (Cat# T6402, Sigma-Aldrich) antibody in 5% bovine serum albumin (BSA) overnight, and Cy3 AffiniPure secondary antibody in 5% donkey serum for 30 min at room temperature.

### Total cholesterol content determination

Total cholesterol content in neural cultures was determined using the Total Cholesterol Assay Kit (Colorimetric, Cat#: STA-384, Cell Biolabs). Neurons from different groups were dissociated with Accutase for 2 min at 37 °C, and the total cell number from each group was counted before cholesterol preparation. Total cholesterol was extracted from cells after being washed three times with cold PBS using 200 µL of a mixture of chloroform: isopropanol: NP-40 (7:11:0.1). The organic phase was transferred to a new tube after centrifugation at 15,000 x *g* for 10 min. The organic solvent was removed by air drying under vacuum at 50 °C for 1 h. Next, lyophilized lipids were dissolved in 170 µl of 1 × assay diluent with vortexing until the solution was homogenous. Cholesterol standards of different concentrations (250 µM, 125 µM, 62.5 µM, 31.3 µM, 15.6 µM, 7.8 µM, 3.9 µM, 1.9 µM, 1.0 µM, and 0) were prepared with 1x assay diluent. The cholesterol reaction reagent was prepared by diluting the cholesterol oxidase 1:50, HRP 1:50, Colorimetric probe 1:50, and cholesterol esterase 1:250 in 1x assay diluent. 50 µL of the diluted cholesterol standards or samples and 50 µL of the cholesterol reaction reagent were mixed completely in a 96-well plate. The covered plate was incubated for 45 min at 37 °C. The plate was then read using a BioTek FLX800 microplate reader at 570 nm. Concentrations of cholesterol per million cells was calculated by comparison to the cholesterol standard curve.

### Cholesterol efflux measurement

Cholesterol effluxes from regular neural cultures, astroglial cells and enriched neurons were determined using the Cholesterol Efflux Fluorometric Assay Kit (Cat#: K582, BioVision). Cholesterol efflux was assessed as per the manufacturer’s instructions, with some modifications. For determining cholesterol efflux, samples were treated as follows: 5 µM GW3965-treated regular neural cultures for 3 days, 1 µM GW3965-treated enriched neurons for 3 days, and 1 µM GW3965-treated astroglial cells for 3 days. Cells were labeled with a mixture of 66.6 µl DMEM/F12 media, 16.7 µl Labeling Reagent, and 16.7 µl Equilibration Buffer containing Reagent A and B/well for 8 h in a 37 °C incubator containing 5% CO_2_. Background fluorescence was determined after incubation for 8 h with a mixture of 66.6 µl of DMEM/F12 medium and 33.4 µl Equilibration Buffer containing Reagents A and B. After labeling, cells were washed with DMEM/F12 media and then incubated with DMEM/F12 media for 6 h in a 37 °C incubator containing 5% CO_2_. At the end of the incubation, the supernatant was transferred to a 96-well plate and fluorescence was measured at Ex/Em = 482/515 nm. Cells were treated with 100 µl Cell Lysis Buffer, and the plate was shaken for 30 min at room temperature. Dissolved cell debris was transferred to a 96-well plate, and fluorescence was determined at Ex/Em = 482/515 nm using a BioTek FLX800 microplate reader. Cholesterol efflux = fluorescence intensity of the medium/(fluorescence intensity of the cell lysate + medium), performed in triplicate for each group.

### ApoE content in regular neural culture and enriched astroglia-conditioned medium

Conditioned medium from 10-week WT, ATL1-P342S, H9, and ATL1-A161P #70 regular neural cultures was prepared and concentrated. Similarly, conditioned medium from enriched astroglial cell cultures was collected. After collecting conditioned medium, proteins of the wild-type, ATL1-P342S, H9, and ATL1-A161P #70 cultures were solubilized using RIPA buffer (Thermo Scientific) with PMSF protease inhibitor (Thermo Scientific) and Halt Protease Inhibitor Cocktail (Thermo Scientific). Conditioned medium was collected and centrifuged at 1000×*g* for 4 min and then concentrated fivefold using Amicon Ultra-4 filter devices (10 kD). ApoE content was measured in concentrated WT, ATL1-P342S, H9, and ATL1-A161P #70 neural culture-conditioned media using a Human ApoE ELISA Kit (Cat#: EHAPOE, Invitrogen) as per the manufacturer’s instructions. Briefly, 100 µL of ApoE standard and conditioned media were added to anti-human ApoE precoated wells of strip plates. Wells were covered and incubated for 2.5 h. After washing with 1 × wash buffer four times, wells were incubated with 100 µL of 1 × prepared biotinylated antibody for 1 h. Then, the solution was discarded and 100 µL of prepared Streptavidin-HRP solution was added to each well. This solution was discarded after incubation for 45 min and 100 µL of TMB substrate was added. Wells were placed in the dark for 30 min, and 50 µL of stop solution was added to each well. The plate was evaluated immediately using an ELISA plate reader (BioTek) set at 450 nm and 550 nm. The 550 nm values were subtracted from 450 nm values to correct for optical imperfections in the microplate. A standard curve was generated, and results were calculated manually.

### Immunocytochemistry

Neural or astroglial cultures on coverslips were washed with ice-cold PBS, followed by incubation in ice-cold 4% paraformaldehyde (Sigma-Aldrich) in PBS for 20 min. After washing 3 times with PBS for 5 min each, cell cultures were incubated with 0.2 Triton X-100 (Sigma-Aldrich) solution for 10 min to permeabilize the cells, followed by washes with PBS. Samples were blocked with 10% donkey serum in PBS for 1 h and then incubated with primary antibodies diluted in the blocking solution (5% donkey serum and 0.1% Triton X-100 in PBS) overnight at 4 °C. After 4 washes with PBS for 10 min each, samples were incubated with secondary antibodies conjugated to Alexa Fluor 488, Cy3 AffiniPure secondary antibody in 3% donkey serum for 30 min at room temperature. After washing with PBS, coverslips were incubated with Hoechst for 5 min, washed, and then mounted with Fluoromount-G (Southern Biotech).

Primary antibodies used in this study were: rat anti-Ctip2 (Cat# ab18465, Abcam), mouse anti-GFAP (Cat# 75-240, NeuroMab), rabbit anti-Tau (Cat# T6402, Sigma), and mouse anti-β-tubulin (Cat# E7, DSHB, 1:100). For immunostaining of GW3965-treated neural cultures or astroglial cultures, 5 µM of GW3965 was used for 7 days in measuring axonal swellings (Tau staining). At least three coverslips for each group were used for immunostaining, and at least 5 fields in each coverslip were imaged using an Olympus confocal microscope or Olympus IX83 microscope. Axonal outgrowth was measured using Fiji software.

### Visualization and quantification of LDs in neurons and astroglial cells

8-week cortical PNs and 6-month astroglial cells were plated on poly-ornithine and Geltrex-coated coverslips. To determine the effects of GW3965 on LDs in astroglial cells, astroglial cells were treated with 1 µM of GW3965 for 3 days, with DMSO as a vehicle control. Neurons and astroglial cells were collected and immunostained as described above. βIII-tubulin and GFAP primary antibodies were used to identify neurons and astroglial cells, respectively. Before staining nuclei with Hoechst, 0.1 µg/mL of LD540 was added to the cells for 10 min to label LDs. At least three coverslips for each group were used for immunostaining, and at least 5 fields in each coverslip were imaged using an Olympus confocal microscope. LD size and numbers of LDs per cell were quantified using Fiji software.

### Real-time quantitative PCR

Total RNA samples were isolated from regular neural cultures or astroglial cells using TRIzol (Invitrogen). 1 µg of RNA was used to generate cDNA using the High-Capacity cDNA Reverse Transcription Kit (Applied Biosystems). Real-time PCR was performed using the PowerUp SYBR Green Master Mix (Applied Biosystems) in the QuantStudio 6 Flex Real-Time PCR System (Applied Biosystems). PCR cycling conditions were: 50 °C for 2 min, 95 °C for 3 min, 45 two-step cycles at 95 °C for 15 s and 60 °C for 60 s, followed by melt-curve stage at 95 °C for 15 s, 60 °C for 60 s, and 95 °C for 15 s. Details of qRT–PCR primers are listed in Additional file 1: Table S1.

For determining effects of GW3965 on cholesterol metabolism-associated gene expression in ATL1-P342S and ATL1-A161P #70 regular neural cultures, D49 neural cultures were treated with 5 µM GW3965 or DMSO (control) for 3 days. For astroglial cells, 6-month-old astroglial cells were treated with 1 µM GW3965 or DMSO (control) for 3 days. Triplicate reactions were performed for real-time PCR.

### Synaptophysin transport

Synaptophysin transport along axons was performed after the neurons were treated with CellLight™ Synaptophysin-RFP (BacMam 2.0, Cat#: C10610, Invitrogen) for 2 days. Synaptophysin-RFP is a fusion construct of synaptophysin and TagRFP, and it is packaged in the insect virus baculovirus. Synaptophysin-RFP particles are provided as 1 × 10^8^ particles/mL solution in the kit. Per the manufacturer’s protocol, we added 10 µL of the solution including 1 × 10^6^ particles in 24-well plates and incubated these with cells for 48 h. The efficiency of transduction for human neurons is around 10%, which is sufficient to examine axonal transport and is comparable to a previous study [63]. After treatment, synaptophysin transport within axons was assessed through live-cell imaging using an Olympus IX83 microscope equipped with an incubation chamber. Neural cultures were maintained at 37 °C with 5% CO_2_ in the incubation chamber. Axons were identified according to morphological criteria that included constant thin diameter, long neurites with no branching, and direct emergence from the neuronal cell body [10]. Synaptophysin transport within axons was captured every 5 s for a total duration of 5 min, yielding 60 frames. At least 5 locations were randomly selected from each of three replicate coverslips for each group. Quantifications were performed using Fiji software with plugins of ‘Macros’ and ‘Multiple Kymograph’ following a protocol described previously [40]. Images were exported as an item of ‘tiff’ series, and axons were selected using ‘segmented line’ in Fiji software. Images were converted to 2D kymographs using the ‘Multiple Kymograph’ plugin. Within the kymographs, average anterograde and retrograde moving speeds were analyzed using the ‘Macros’ plugin. The anterograde-moving synaptic vesicle (SV) number, retrograde-moving SV number, and moving SV velocities were analyzed from kymographs as described [55]. SVs moving < 5 µm were classified as static SVs, and SVs moving ≥ 5 µm were considered to be moving SVs. The net moving distance was calculated by subtracting the average distance per SV moving anterogradely or retrogradely (anterograde $\varSigma {\text{dx}}$–retrograde $\varSigma {\text{dx}}$). For determining the effect of GW3965 on synaptophysin transport of WT, ATL1-P342S, and ATL1-A161P #70 cortical PNs, regular neural cultures were treated with 5 µM GW3965 or DMSO (control) for 3 days before live-cell imaging. Cells were incubated in NDM with 5 µM GW3965 or DMSO (control) during the live-cell imaging.

### Mitochondrial transport

To examine the effects of cholesterol, ATL1-P342S and ATL1-A161P #70 cortical PNs were supplemented with 10 µg/mL cholesterol (Sigma-Aldrich) in culture for 7 days. On the 8th day following treatment, mitochondrial transport within axons was assessed through live-cell imaging using an Olympus IX83 microscope equipped with an incubation chamber. Neurons were stained with 50 nM MitoTracker Red CMXRos (Cat#: M7512, Invitrogen) for 3 min to visualize mitochondria and then incubated in fresh NDM for live-cell imaging. Neurons were kept at 37 °C with 5% CO_2_ during imaging. Pictures were captured every 5 s for 5 min. Quantifications were performed using ImageJ with Macros and Multiple Kymograph as described previously [45]. GM1 and H9 cell line-derived neurons were used as controls. Anterograde or retrograde moving mitochondria were defined as moving 5 µm forward or backward from the origin during the entire period (5 min).

### mRNA sequencing

We performed RNA-seq experiments on both cortical neurons and spinal motor neurons derived from iPSCs. Cortical PNs and spinal motor neurons were differentiated from the same batch of iPSCs in parallel to minimize variations. Both WT control iPSCs (GM1) and SPG3A-patient iPSCs were differentiated into cortical PNs (forebrain) and spinal motor neurons (spinal) based on protocols established in our laboratory [35, 36]. Week 7 control and SPG3A forebrain and spinal neuron samples were collected. RNA was isolated from week 7 control and SPG3A forebrain and spinal neuron cultures using TRIzol reagent (Invitrogen) following the manufacturer’s instructions. Libraries were prepared using Illumina’s specifications for polyA-plus stranded reactions. Samples were analyzed using an Illumina HiSeq2000 sequencer. Reads were mapped to the human hg19 genome using TopHat2 and Bowtie2. Expression quantification in fragments per kilobase gene model per million base pairs (FPKM) was performed using Cuffdiff [70]. The data were deposited in the ArrayExpress database (accession number E-MTAB-7770).

### Statistical analysis

All data are presented as mean ± SD or SEM. Statistical parameters including statistical value, statistical significance (*p* value), SD or SEM, are detailed in the figures and figure legends. For statistical significance in mean values between two groups, we performed Student’s *t* test. Statistical significance of mean values among multiple sample groups was analyzed using ANOVA. Chi square analysis was used to analyze differences in the sizes of LDs. *p* < 0.05 was considered significant.

## Results

### *ATL1* mutation results in axonal defects specifically in cortical PNs

**Fig. 1:**
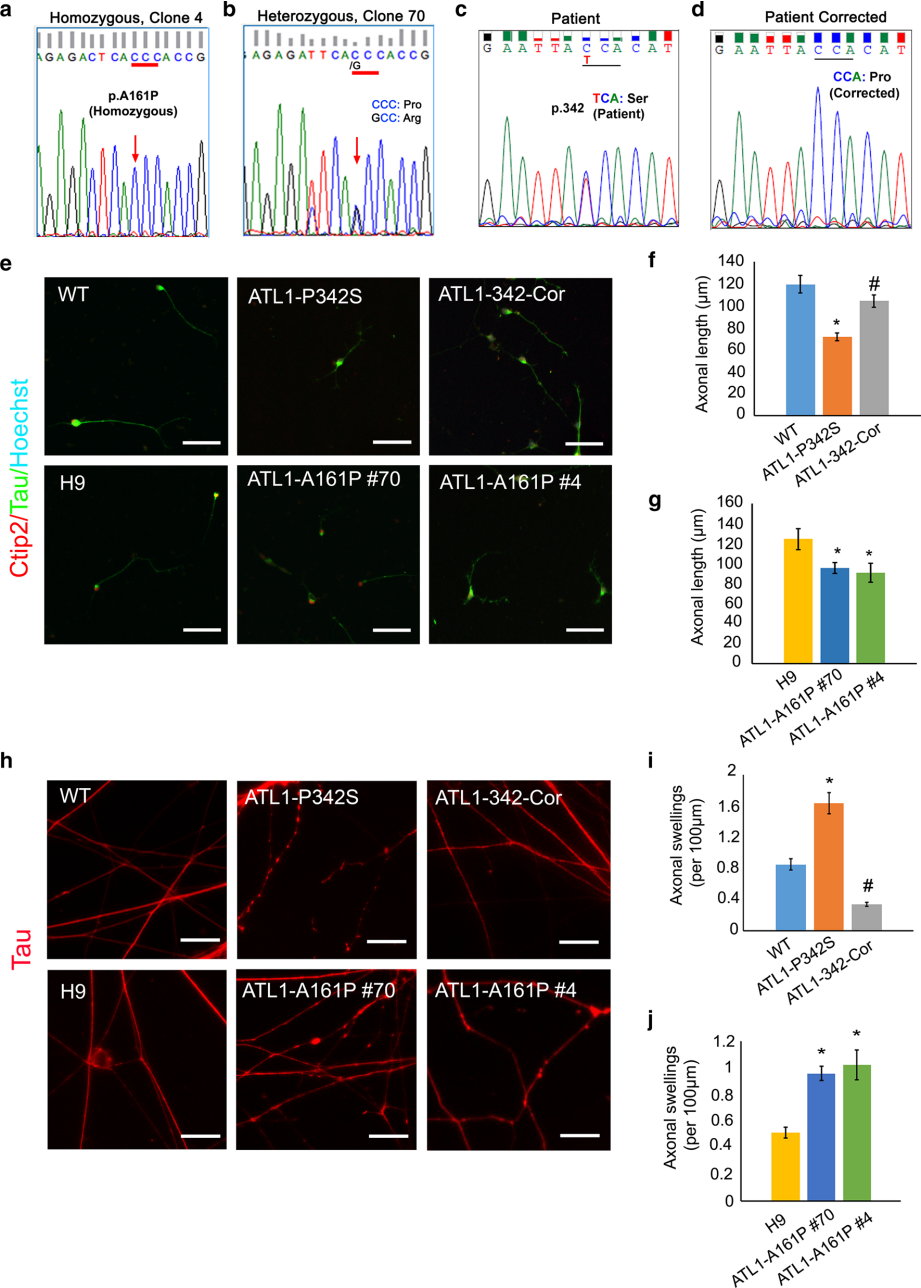
SPG3A mutations in *ATL1* result in axonal outgrowth defects in isogenic hPSC lines. **a**, **b** Sequencing of genomic *ATL1* confirms validity of both homozygous (**a**; ATL1-A161P #4) and heterozygous clones (**b**; ATL1-A161P #70). **c**, **d** Correction of point mutations in SPG3A iPSCs to generate isogenic WT iPSCs. Sequencing of genomic *ATL1* locus in SPG3A patient-derived iPSCs (ATL1-P342S) (**c**), which is corrected to WT (ATL1-342-Cor) (**d**). **e** Representative images of Tau staining (green) assessing axonal outgrowth of cortical PNs in WT, ATL1-P342S, ATL1-342-Cor, H9, ATL1-A161P #70 and ATL1-A161P #4 groups. Ctip2 (red); Hoechst (cyan). Scale bar: 50 µm. **f**, **g** Quantification of axonal length in cortical PNs derived from WT, ATL1-P342S, ATL1-342-Cor, H9, ATL1-A161P #70 and ATL1-A161P #4 hPSCs. **h** Tau staining reveals axonal swellings in WT, ATL1-P342S, ATL1-342-Cor, H9 and ATL1-A161P cortical PNs at 12 weeks after differentiation. Scale bar: 20 µm. **i**, **j** Quantification of axonal swellings per 100 µm of axon length in WT, ATL1-P342S, and ATL1-342-Cor, H9 and ATL1-A161P cortical PNs. Data are represented as mean ± SEM from triplicate biological samples. The statistical significance of mean values among three groups was analyzed using ANOVA. *p < 0.05 compared to WT (for ATL1-P342S) and H9 (for ATL1-A161P #70 and #4), respectively; ^#^*p* < 0.05 compared to ATL1-P342S

SPG3A patients exhibit early-onset spasticity in leg muscles, which is caused by altered neurotransmission to muscles from cortical PNs [47]. To determine whether mutant *ATL1* impairs axonal development of cortical PNs, we generated isogenic cell lines by knocking-in the A161P *ATL1* mutation into H9 hESCs using CRISPR-Cas9 (Additional file 2: Fig. S1a). PCR analysis showed the incorporation of ssODN in the cloned cells (Additional file 2: Fig. S1b). The isogenic homozygous line ATL1-A161P #4 and heterogeneous line ATL1-A161P #70 with the c.481G > C substitution were validated by DNA sequencing (Fig. 1a and b). ATL1-A161P mutant cells maintained normal karyotypes after multiple passages (Additional file 2: Fig. S1c). In addition, we corrected the heterozygous *ATL1* P342S pathogenic SPG3A mutation in iPSCs we previously generated from patient-derived cells [77], yielding another isogenic pair (Fig. 1c and d) referred to ATL1-P342S and ATL1-342-Cor iPSCs.

Isogenic hPSCs were differentiated into cortical PNs following published methods [36] which involved the formation of embryonic bodies (EBs, also known as stem cell aggregates), the specification of neuroepithelial cells with apparent rosettes structures, and the differentiation of cortical PNs (Additional file 2: Fig. S1d and S1e). The cortical PN identity of the differentiated cells was confirmed by positive immunostaining for Tau, an axonal marker, and Ctip2, a transcription factor enriched in subcerebral cortical PNs, 2 days after plating the neural progenitors (total D42 post hPSC differentiation) (Fig. 1e). Measurement of Tau^+^ neurites showed a significant reduction in neurite length in *ATL1* mutant (i.e., ATL1-P342S, ATL1-A161P #70 and ATL1-A161P #4) cortical PNs compared to the corresponding isogenic control cortical PNs (Fig. 1f and g). No significant differences were observed between homo- and heterozygous mutant ATL1-A161P cells (Fig. 1g). Moreover, neurite lengths of ATL1-342-Cor cortical PNs were similar to those of control cells, indicating that proper axonal elongation was restored when SPG3A mutations in *ATL1* were corrected in cortical PNs (Fig. 1f). These results establish that *ATL1* mutation underlies the decreased axonal outgrowth during axonal development in cortical PNs.

To determine if the axonal defect is specific to cortical PNs, we generated both spinal motor neurons and forebrain (cortical PN) neurons from control and SPG3A iPSCs (Additional file 2: Fig. S2a). These regional progenitors were specified at 4 weeks after hPSC differentiation [36]. These progenitors were cultured in suspension and then dissociated, plated on coverslips, and subjected to examination of neurite outgrowth at 2 days after plating (total 6 weeks). We found reduced axonal outgrowth in SPG3A cortical PNs, but not spinal motor neurons (Additional file 2: Fig. S2b), recapitulating HSP cell-type-specific defects in patient iPSC-derived neural cultures.

A characteristic pathological change in numerous HSP models, both in animals and patient-derived cells, is a marked increase in axonal swellings [18, 20, 29]. With prolonged culture (total 12 weeks), we found that the Tau^+^ axons of cortical PNs derived from SPG3A iPSCs were thinner and exhibited many more areas of enlargement (swellings) than the isogenic corrected cells (Fig. 1h); the number of axonal swellings per axonal segment was significantly increased in ATL1-P342S cortical PNs as compared to those derived from both wild-type (WT) and isogenic control iPSCs (Fig. 1i). Similarly, the number of axonal swellings was significantly increased in ATL1 A161P cortical PNs (Fig. 1j). Thus, our cultured cortical PNs recapitulate axonal defects seen in HSP patients.

### *ATL1* mutation impairs axonal transport in human cortical PNs

**Fig. 2:**
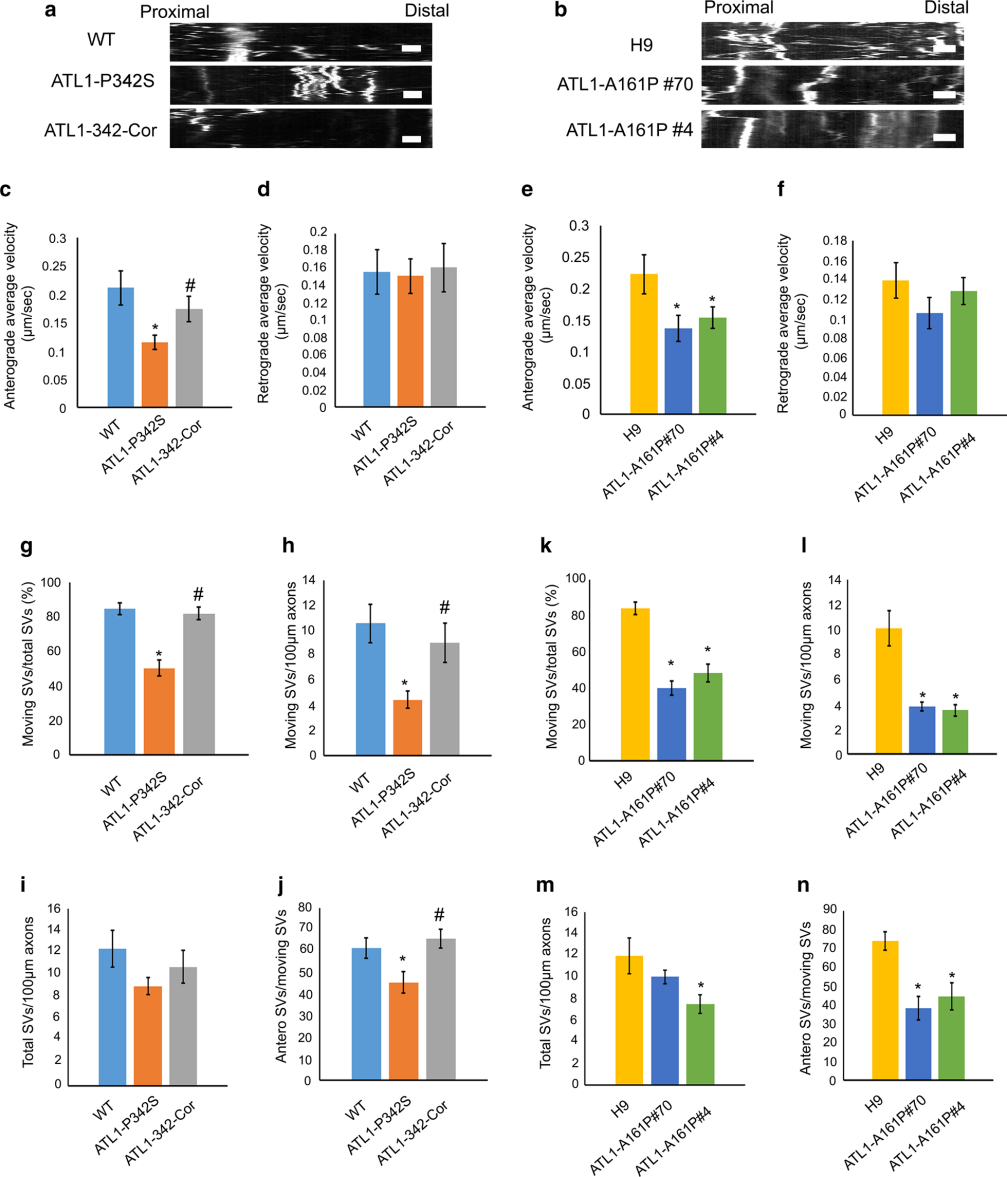
Impaired axonal transport of synaptophysin in *ATL1* mutant cortical PNs derived from isogenic hPSCs. **a**, **b** Kymographs representing synaptic vesicles (SVs) transport along axons of WT, ATL1-P342S, ATL1-342-Cor, H9, ATL1-A161P #70 and ATL1-A161P #4 cortical PNs infected with synaptophysin::RFP virus. The x-axis indicates axon length from proximal to distal areas. The y-axis indicates time-lapse duration in min (y = 5 min). Scale bar: 5 µm. **c** Anterograde and **d** retrograde average moving velocities of SVs along axons in WT, ATL1-P342S, and ATL1-342-Cor cortical PNs. **e** Anterograde and **f** retrograde average moving velocities of SVs along axons in H9, ATL1-A161P #70, and ATL1-A161P #4 cortical PNs. **g**, **h** Graphs show the ratios of moving SVs to total SVs (**g**) and moving SVs per 100 µm of axon (**h**) in WT, ATL1-P342S, and ATL1-342-Cor cortical PNs. **k**, **l** Graphs represent the ratio of moving SVs in relation to the total number of SVs (**k**) and the ratio of moving SVs per 100 µm of axon (**l**) in H9, ATL1-A161P #70 and ATL1-A161P #4 cortical PNs. **i**, **j** Number of total SVs per 100 µm of axon (**i**) and the ratio of anterograde moving SVs in relation to moving SVs (**j**) in WT, ATL1-P342S and ATL1-342-Cor cortical PNs. **m**, **n** Number of total SVs per 100 µm of axon (**m**) and the ratio of anterograde moving SVs in relation to moving SVs (**n**) in H9, ATL1-A161P #70 and ATL1-A161P #4 cortical PNs. Data are represented as mean ± SEM from 3 independent samples. The statistical significance of mean values among three groups was analyzed using ANOVA. *p < 0.05 compared to WT (for ATL1-P342S) and H9 (for ATL1-A161P #70 and #4), respectively. ^#^*p* < 0.05 compared to ATL1-P342S

Neurons are highly polarized and require efficient transport of molecules and organelles along axons to perform their normal functions [39]. Impaired axonal transport is an early and critical pathology during processes of axonal degeneration [59]. We previously showed that axonal transport of mitochondria is impaired in SPG3A cortical PNs [77]. To examine whether axonal transport functions are impaired more generally, we analyzed the transport of synaptophysin in cortical PN axons, since synaptophysin is considered a reliable marker for examining axonal defects [26, 69]. Using live cell imaging of cells infected with RFP::synaptophysin virus, we observed a significant reduction in the average velocity of anterograde synaptophysin transport in ATL1-P342S cortical PNs as compared to WT cortical PNs (Fig. 2a and c). The retrograde moving velocity, however, was not affected in ATL1 P342S cortical PNs (Fig. 2d). Importantly, correction of the *ATL1* mutation reversed the decrease in anterograde velocity of synaptophysin-RFP cargos (Fig. 2a and c). Similar results were observed in *ATL1* mutant knock-in hESC-derived cortical PNs, with a significant reduction in anterograde synaptophysin transport in both ATL1-A161P #70 and #4 cortical PNs as compared to H9 cortical PNs (Fig. 2b and e). The retrograde velocities were not significantly altered in either the homozygous (ATL1-A161P #4) or heterozygous (ATL1-A161P #70) *ATL1* mutant cells (Fig. 2b and f). These findings indicate that mutations in *ATL1* result in reduced axonal anterograde transport velocity.

In addition to the transport velocity, we analyzed the movement events of synaptic vesicles (SVs, indicated by synaptophysin) (Fig. 2g–n). The percentage of moving SVs among total SVs, moving SVs per 100 µm axon, and the percentage of anterograde SVs among moving SVs were all reduced in ATL1-P342S cortical PNs as compared to WT cortical PNs (Fig. 2g, h and j). In contrast, total SVs per 100 µm axon in ATL1-P342S cortical PNs were not altered (Fig. 2i). The reduced SV moving events in ATL1-P342S cortical PNs were efficiently rescued when the *ATL1* mutation was corrected (Fig. 2g, h and j). Significant defects in synaptophysin transport were also found in cortical PNs with the other *ATL1* mutation (ATL1-A161P) as compared to H9 cortical PNs (Fig. 2k–n). Both ATL1-A161P #70 and ATL1-A161P #4 cortical PNs exhibited significant reductions in anterograde SVs (Fig. 2n) and the number of moving SVs (Fig. 2k and l). These data revealed that missense mutations in *ATL1* result in defective SV movement, especially anterograde transport, in SPG3A cortical PNs, and the impairments are rescued when the SPG3A mutation is corrected. Given that there were no significant differences in phenotypic defects between homozygous and heterozygous *ATL1*-mutant iPSC-derived cortical PNs, the heterozygous mutant line (ATL1-A161P#70) was utilized in the following experiments.

### *ATL1* mutation results in impaired cholesterol homeostasis in SPG3A cortical PNs

How mutant *ATL1* results in axonal and synaptic defects selectively in cortical PNs remains largely unclear [78]. To address this question, we performed mRNA-sequencing to compare gene expression profiles between WT and SPG3A neural cell cultures at 7 weeks. At this stage, cultures consisted of mostly neurons and a small number of astrocytes. Given the cell type-specific axonal changes, we generated spinal motor neurons as well as cortical PNs from the same PSCs for gene expression profiling (Additional file 2: Fig. S2a). Genes that are changed in SPG3A cortical PNs were identified by comparing cortical PNs derived from WT and SPG3A iPSCs (Additional file 2: Fig. S2c). A threshold of genes with a minimal FPKM > 1 and absolute value of log-fold change > 1 (so gene FPKM either increased or decreased by a factor of at least 2) were chosen for further analysis. To identify genes that are specifically changed in cortical PNs, spinal neurons derived from control and SPG3A iPSCs were also compared. Changes that occurred in both cortical PNs and spinal neurons were excluded, which resulted in the identification of 956 genes reduced specifically in SPG3A forebrain cells, and 227 genes that were increased. Using gene ontology (GO) analysis with DAVID, we examined the altered genes. The most significantly changed pathways were cell–cell adhesion, canonical Wnt signaling, and cell migration (Additional file 3: Table S2). Some common pathways including cell proliferation and cell matrix adhesion were significantly altered as well (Additional file 3: Table S2). Strikingly, lipoprotein metabolic processes were one of the top candidate pathways altered specifically in the cortical cells (Additional file 3: Table S2). Lipoproteins are synthesized in glial cells and are important for lipid and cholesterol transport in the central nervous system.

ER calcium ion homeostasis and cholesterol efflux were significantly altered in cortical cells derived from both SPG3A iPSCs. qRT-PCR validated the reduction of *NR1H2*, *PLTP*, *ABCA2* and *LRP1* in ATL1-P342S and ATL1-A161P #70 cortical PNs (Additional file 2: Fig. S2d). Reduced cholesterol levels have been reported in the serum samples from mice of SPG31, a HSP subtype caused by mutations in the *Reep1* gene encoding REEP1 protein that can bind with the SPG3A protein [64]. These suggest that impaired cholesterol metabolism and trafficking may be involved in HSP pathogenesis.

**Fig. 3:**
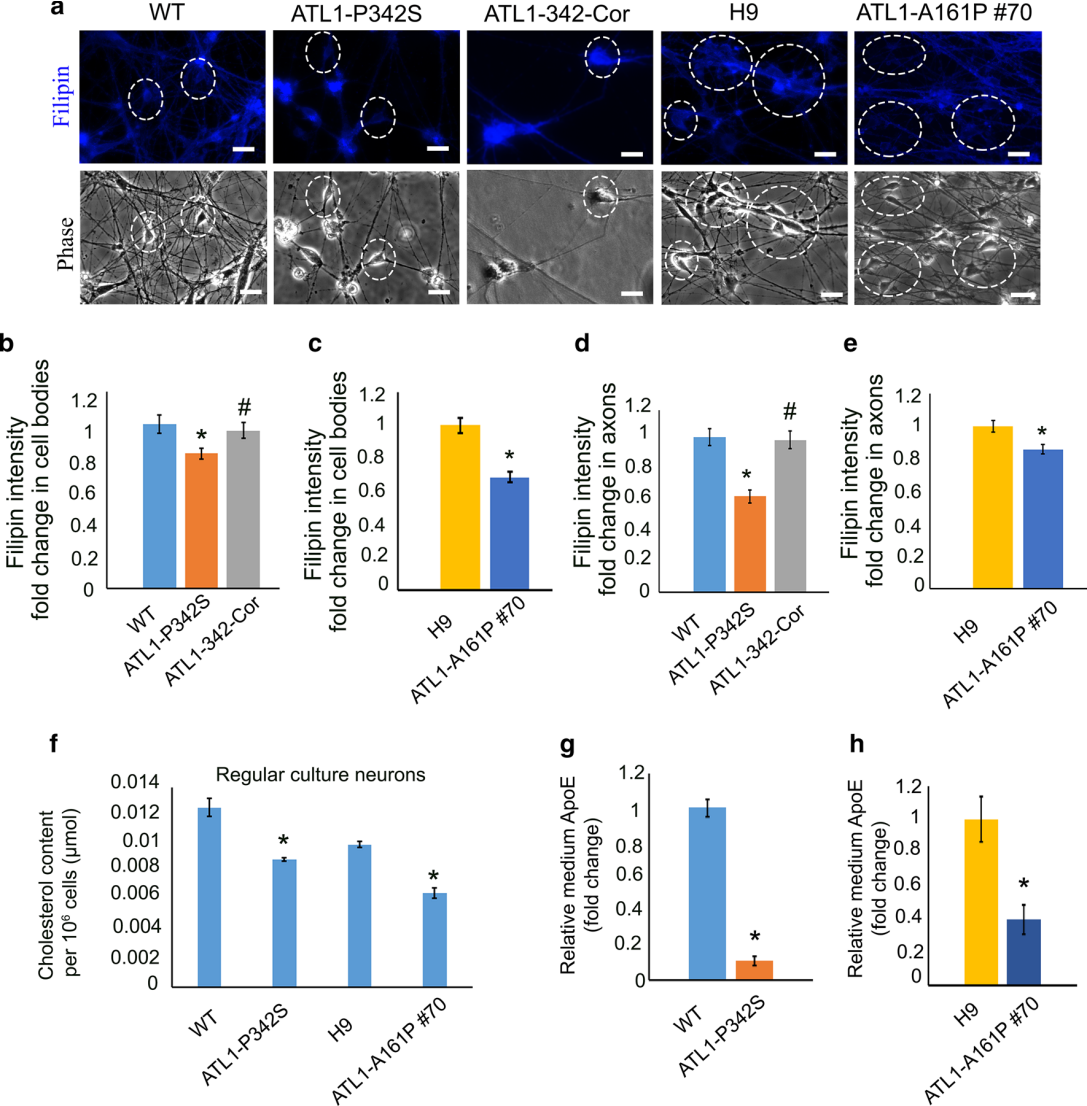
Defects of cholesterol metabolism in *ATL1* mutant cortical PNs regular cell culture. **a** Representative Filipin III (blue) staining and corresponding phase images for WT, ATL1-P342S, ATL1-342-Cor, H9 and ATL1-A161P #70 cortical PNs in regular neural culture. The white circle highlights the cortical neurons in regular cell culture. Scale bar: 20 µm. **b**–**e** Filipin intensity fold-change in cell bodies (**b**) and axons (**d**) in WT, ATL1-P342S, and ATL1-342-Cor cortical PNs quantified by ImageJ. Filipin intensity fold-change in cell bodies (**c**) and (**e**) axons of H9 and ATL1-A161P #70 cortical PNs. **f** Cholesterol content in WT, ATL1-P342S, H9 and ATL1-A161P #70 regular neural cultures determined by a colorimetric method. **g** Relative ApoE content in culture medium of 10-week cortical PNs derived from WT and ATL1-P342S cells. There is a significant reduction in ApoE content in ATL1-P342S cortical PNs. **h** Significant reduction of ApoE content was also observed in ATL1-A161P #70 cortical PN cultures when comparing to WT cultures. Data are represented as mean ± SEM from 3 independent samples. *p < 0.05 for ATL1-P342S compared to WT and for ATL1-A161P #70 compared to H9. ^#^*p* < 0.05 compared with ATL1-P342S

Cholesterol plays an essential role in neuronal activity and is associated with neurodegeneration [16, 41, 66]. To dissect the role of cholesterol in SPG3A, we first examined cholesterol levels in SPG3A cultures. Filipin staining, a measure of cholesterol content, was significantly decreased in ATL1-P342S neuronal cell bodies and axons compared to isogenic neurons (Fig. 3a, b, d and Additional file 2: Fig. S3). When the *ATL1* mutation in ATL1-P342S was corrected, the Filipin staining intensity was increased in cell bodies and axons to a level comparable to that in control cortical PNs (Fig. 3a, b and d). Similarly, the significant decrease in cholesterol intensity was found in ATL1-A161P #70 neuronal cell bodies and axons as compared to H9 neurons (Fig. 3a, c and e). The decreased cholesterol content in mutant cortical PN cultures was further verified by colorimetric measurement using the Total Cholesterol Kit (Fig. 3f).

One of the main cholesterol sources for neurons is the uptake of exogenous cholesterol that is synthesized in glial cells [21, 41]. This is particularly important for neurons after birth, when neurons gradually lose their ability to synthesize cholesterol and are more dependent on glial cells to obtain cholesterol. The transfer of cholesterol from glial cells to neurons is mediated by lipoproteins, especially ApoE, in the brain. ApoE is synthesized primarily in glial cells, but not neurons [25, 58]. In our cultures, in addition to neurons, there are about 15% GFAP^+^ astrocytes (Additional file 2: Fig. S4) that could be the source for ApoE. Using the Apolipoprotein E Human ELISA kit, we detected a significant reduction of ApoE in the medium from both ATL1-P342S and ATL1-A161P cortical PN cultures (Fig. 3g and h), which dovetails with our mRNA sequencing data, suggesting impaired lipoprotein metabolism in SPG3A cultures. Taken together, our results reveal impaired cholesterol homeostasis and lipoprotein metabolism in SPG3A cortical neural cells.

### Axonal defects of *ATL1* mutant cortical PNs are mitigated by restoring cholesterol levels

**Fig. 4:**
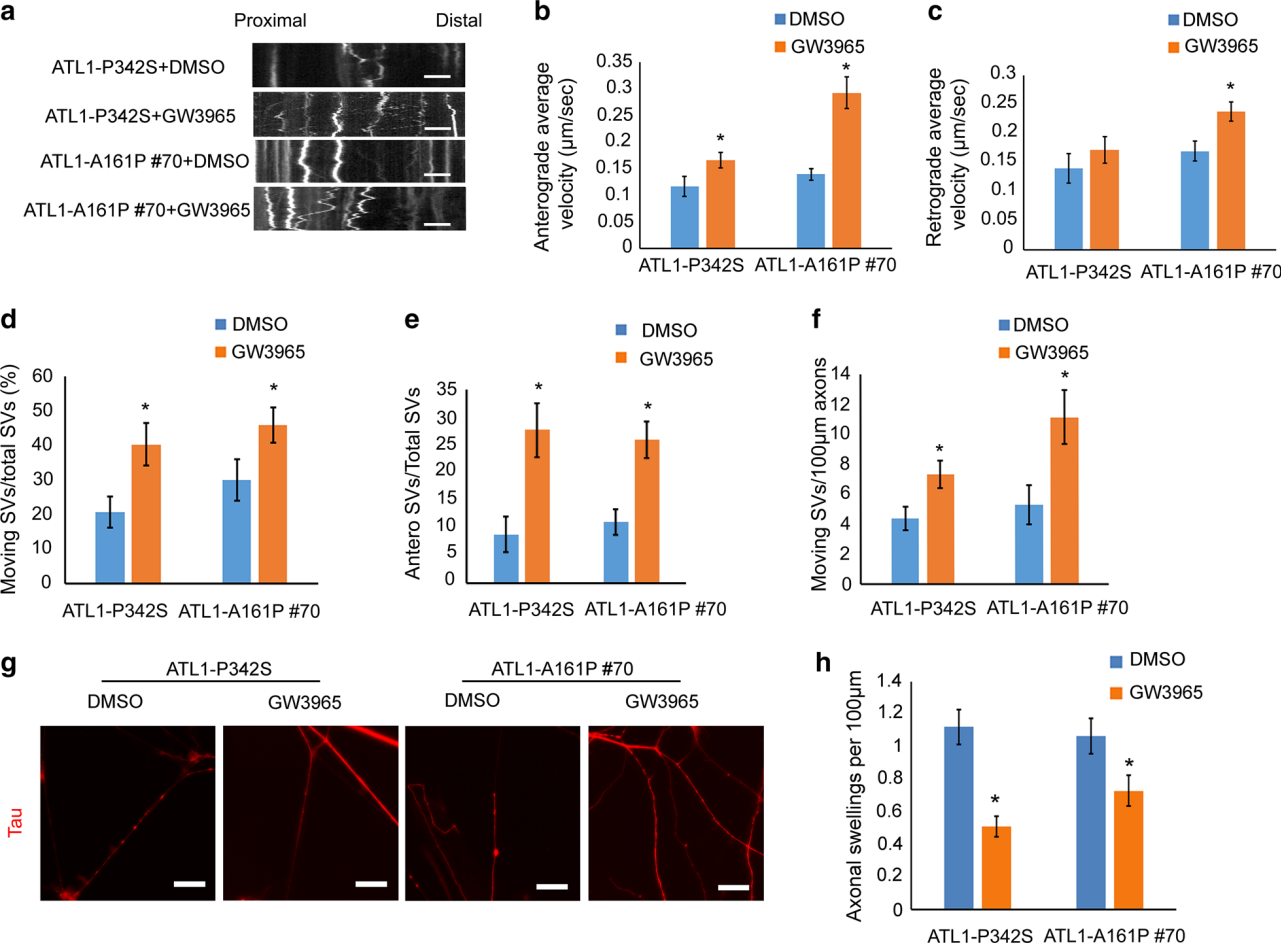
GW3965 treatment mitigates axonal defects in *ATL1* mutant cortical PNs. **a** Synaptophysin transport in ATL1-P342S and ATL1-A161P #70 cortical PNs after treatment for 3 days with 5 µM GW3965 or DMSO. The x-axis indicates the axon length from proximal to distal areas. The y-axis indicates time-lapse duration in min (y = 5 min). Scale bar: 5 µm. **b**, **c** Anterograde (**b**) and retrograde (**c**) average moving velocities of SVs after GW3965 treatment for 3 days. **d** Ratio of moving SVs in relations to the total number of SVs after GW3965 treatment. **e** Ratio of anterograde moving SVs in relation to total SVs after GW3965 treatment. **f** Ratio of moving SVs per 100 µm of axon after GW3965 treatment. **g** Axonal swellings (Tau, red) of ATL1-P342S and ATL1-A161P #70 cortical PNs after GW3965 treatment for 7 days at D84. Scale bar: 20 µm. **h** Quantifications of axonal swellings of GW3965-treated ATL1-P342S and ATL1-A161P #70 cortical PNs. Data are presented as mean ± SEM from triplicate biological samples. *p < 0.05 compared to DMSO-treated ATL1-P342S or ATL1-A161P #70 cortical PNs using two-sided Student’s *t*-test

Axonal defects and reduced cholesterol levels in *ATL1* mutant cortical PNs suggest that impaired cholesterol homeostasis might mediate SPG3A pathogenesis. To test this hypothesis, we pharmacologically regulated cholesterol trafficking with GW3965, an NR1H2 (Nuclear Receptor Subfamily 1 Group H Member 2) agonist [7]. NR1H2, also known as LXRβ, is a critical regulator of cholesterol trafficking [73]. We observed a significant reduction of *NR1H2* mRNA expression in 7-week SPG3A neural cultures (Additional file 2: Fig. S2d). We then asked whether axonal transport in mutant cortical PNs can be restored by regulating cholesterol homeostasis. Following treatment of these cultures with 5 µM GW3965 [61] or vehicle, axonal transport of synaptophysin was examined. The anterograde synaptophysin transport velocity was significantly improved by GW3965 in ATL1-P342S and ATL1-A161P #70 neurons (Fig. 4a–c). In addition, the moving SVs/total SVs, anterograde SVs/total SVs, and moving SVs/100 µm axons measures were significantly increased when compared to DMSO-treated neurons (Fig. 4d–f). These data indicate that the reduced velocities and movements of synaptophysin transport in SPG3A cortical PN axons can be mitigated by GW3965. Importantly, GW3965 did not significantly alter the transport of synaptophysin in WT cortical PNs (Additional file 2: Fig. S5). Furthermore, axonal swellings per axonal segment in both ATL1-P342S and ATL1-A161P #70 cortical PNs were decreased significantly by GW3965 (Fig. 4g and h), indicating that GW3965 ameliorates the axonal pathology in SPG3A neurons.

**Fig. 5:**
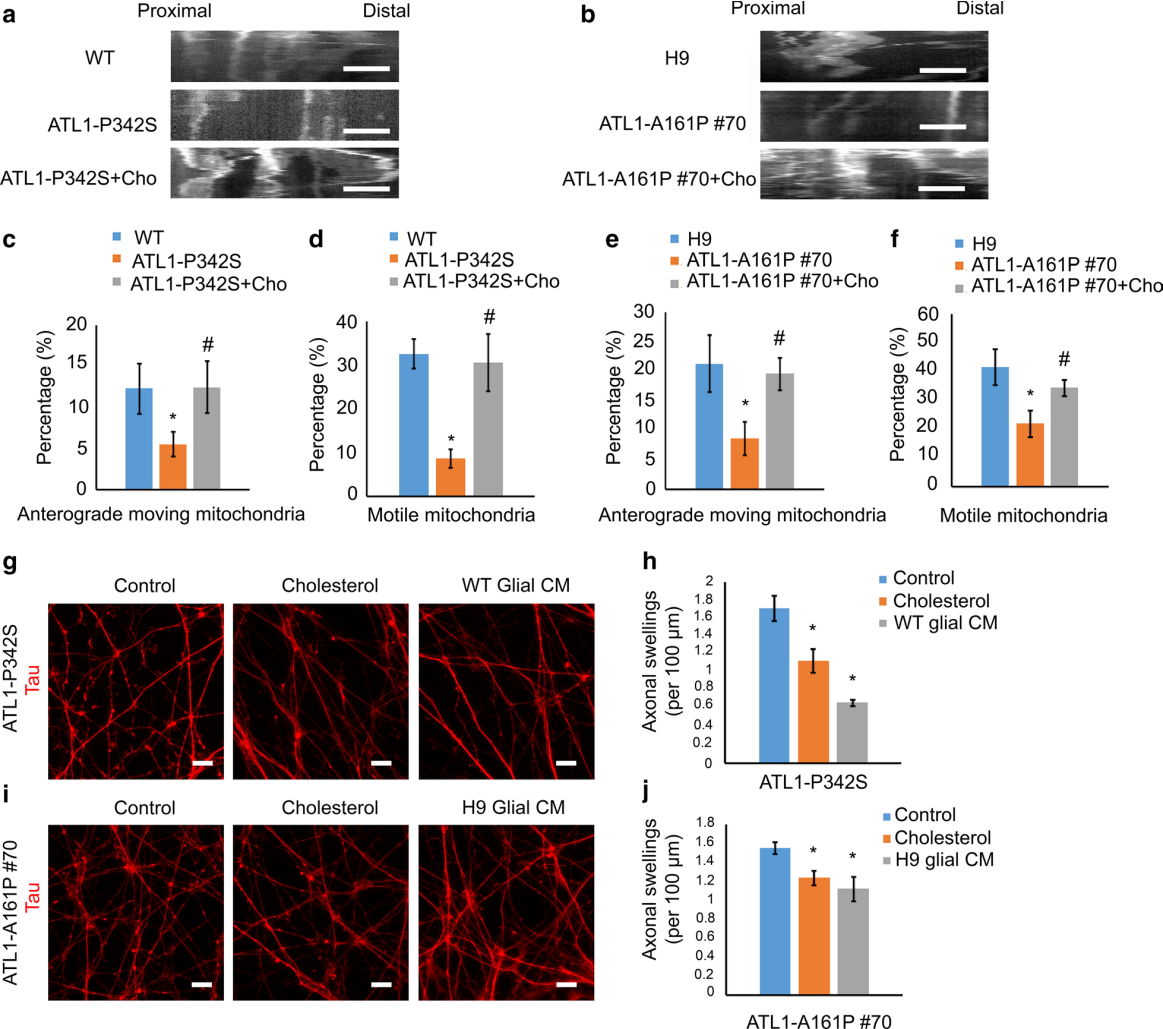
Cholesterol and astroglial cell-conditioned medium rescue axonal transport impairment and suppress axonal swellings in *ATL1* mutant cortical PNs. **a**, **b** Representative kymographs of mitochondrial transport along axons of WT, H9, ATL1-P342S and ATL1-A161P #70 cortical PNs, with or without cholesterol (Cho) treatment as indicated. Scale bar: 5 µm. **c**–**f** Percentage of anterograde moving mitochondria and motile mitochondria in WT, H9, ATL1-P342S, and ATL1-A161P #70 cortical PNs with exogenous Cho treatment. **g**, **i** Representative images of Tau staining indicating axonal swellings of 12-week ATL1-P342S and ATL1-A161P #70 cortical PNs after treatment with cholesterol or glial cell-conditioned medium (CM). WT glial CM for ATL1-P342S neurons and H9 glial CM for ATL1-A161P #70 neurons. Scale bar: 20 µm. **h**, **j** Quantification of axonal swellings per 100 µm of axon in ATL1-P342S and ATL1-A161P #70 cortical PNs with cholesterol or glial CM treatment. WT glial CM for ATL1-P342S neurons and H9 glial CM for ATL1-A161P #70 neurons. Data are represented as mean ± SEM. **c**–**f** *p < 0.05 compared to WT and H9 cells, respectively. ^#^*p* < 0.05 compared to ATL1-P342S or ATL1-A161P #70 by ANOVA. **h**, **j**)*p < 0.05 compared to ATL1-P342S control or ATL1-A161P #70 control by ANOVA

To confirm the role of decreased cholesterol in mediating SPG3A axonal defects, we applied exogenous cholesterol to ATL1-P342S and ATL-A161P #70 cortical PNs. Axonal transport of mitochondria, which is critical for the energy supply of neurons and reduced in SPG3A cortical neurons [77], was examined after cholesterol treatment and compared with control neurons (Fig. 5a and b). The percentage of motile mitochondria, especially in the anterograde direction, was significantly reduced in ATL1-mutated cortical PNs (Fig. 5c–f). Interestingly, cholesterol treatment increased the percentage of motile mitochondria, rescuing axonal transport defects in the SPG3A neurons (Fig. 5c–f). Moreover, the application of cholesterol significantly mitigated the accumulation of axonal swellings in long-term cultures (Fig. 5g–j), suggesting that cholesterol can rescue axonal degeneration in long-term cultures.

Since glial cells are the major source of cholesterol, we examined whether normal glial cells can rescue the axonal defects of SPG3A cortical PNs. SPG3A cortical neural cells were cultured with conditioned medium from normal astroglial cells. The axonal swellings of ATL1-P342S cortical PNs were significantly reduced after treatment with conditioned medium of wild-type astroglial cells (Fig. 5g and h). Similarly, the number of axonal swellings was significantly decreased in ATL1-A161P #70 cortical PNs after treatment with conditioned medium from H9 glial cells (Fig. 5i and j). Taken together, these data demonstrate that impaired cholesterol homeostasis underlies axonal defects of SPG3A cortical PNs, which can be rescued by restoring cholesterol levels via treatment with cholesterol, GW3965 or normal glial cell conditioned medium.

### *ATL1* mutations impair lipid droplet formation in astrocytes

**Fig. 6:**
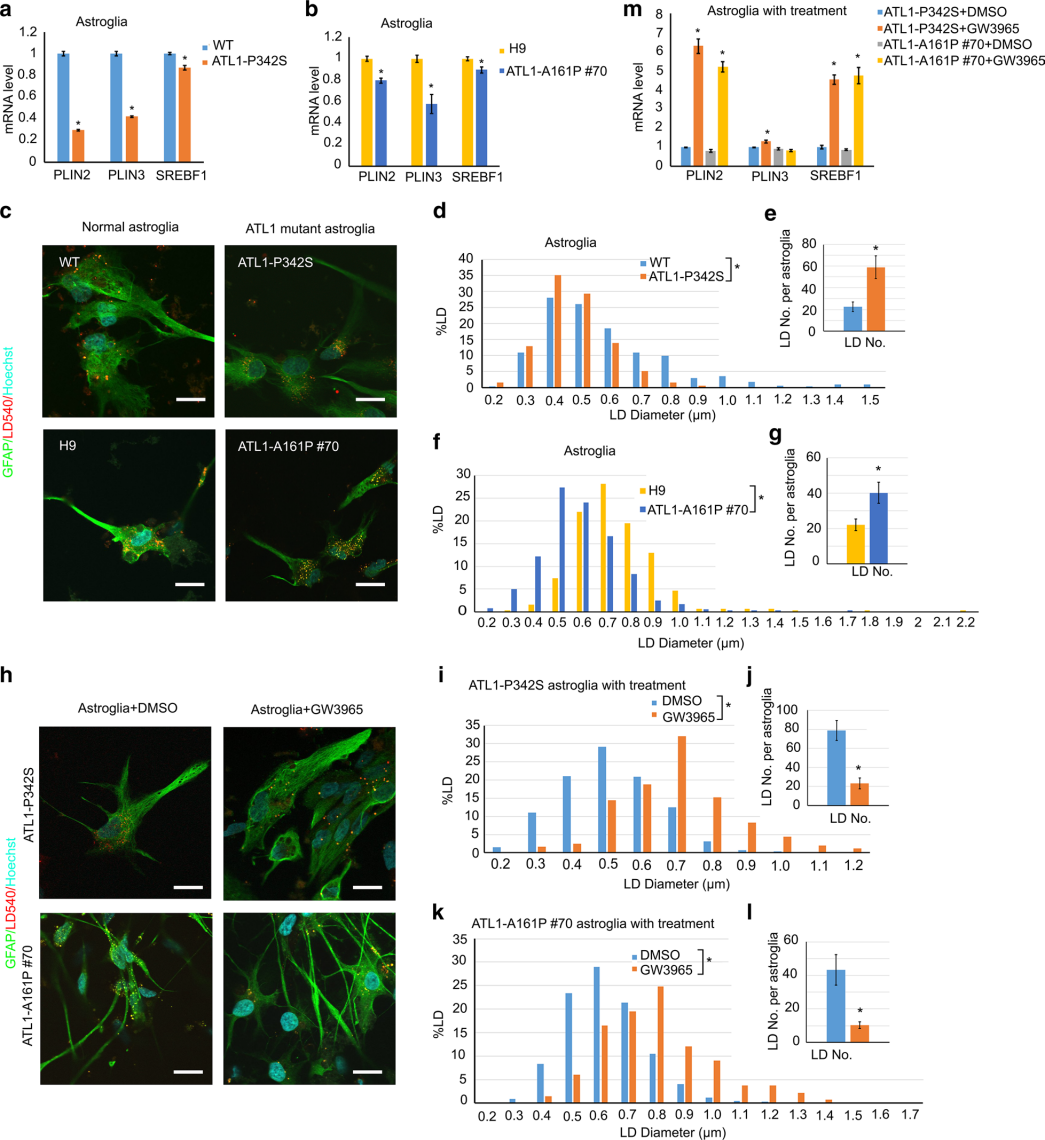
Alterations in LD size and distribution in *ATL1* mutant astroglia. **a**, **b** LD-associated gene expression in WT, ATL1-P342S, H9, and ATL1-A161P #70 astroglia. **c** Visualization of LDs in WT, ATL1-P342S, H9, and ATL1-A161P #70 astroglial cells. **d** Distribution of LD size and **e** LD numbers per astroglial cell in WT and ATL1-P342S astroglia. **f** Distribution of LD size and **g** LDs numbers per astroglial cells in H9 and ATL1-A161P #70 astroglia. **h** Visualization of LDs in ATL1-P342S and ATL1-A161P #70 astroglia after DMSO or 1 µM GW3965 treatment for 3 days. **i** Distribution of LD size and **j** LD number per astroglial cell in ATL1-P342S astroglia after DMSO or 1 µM GW3965 treatment for 3 days. **k** Distribution of LD size and **l** LD number per astroglial cell in ATL1-A161P #70 astroglia after DMSO or 1 µM of GW3965 treatment for 3 days. **m** LD-associated gene expression in ATL1-P342S and ATL1-A161P #70 astroglia after DMSO or 1 µM of GW3965 treatment for 3 days. Red: LD540, green: GFAP, cyan: Hoechst. Scale bar: 20 µm. Data are represented as mean ± SEM. **a**, **b**, **e**, and **g** *p < 0.05 compared to WT or H9 by two-sided Student’s *t*-test. **j**, **l**, and **m** *p < 0.05 compared to ATL1-P342S and ATL1-A161P #70 astroglia after DMSO treatment, respectively. Chi square analysis was used to analyze the difference in the sizes of LDs

Cholesterol is primarily synthesized in glia and transferred to neurons in the mature brain [13, 48]. Our observations that *ATL1* mutant cells exhibit impaired cholesterol homeostasis and that GW3965 (as well as normal glial cell conditioned medium) rescues axonal defects led us to hypothesize that *ATL1* mutations impair cholesterol metabolism in glia, which results in axonal defects. Considering that atlastin-1 can directly modulate LDs [30], we first examined the expression of LD genes. Interestingly, mRNA expression of perilipin 2 (*PLIN2*) and perilipin 3 (*PLIN3*), critical LD genes, was significantly reduced in glial cells (Fig. 6a and b) but not in neurons (Additional file 2: Fig. S6b and S6c). We then examined the size and distribution of LDs in neurons and glial cells derived from SPG3A and control hPSCs using LD450 staining. Strikingly, we found numerous LDs in astrocytes (Fig. 6c), while there were very few LDs in enriched neuronal cultures (Additional file 2: Fig. S6a). Further analysis of glial cell cultures revealed that the size of LDs was significantly reduced in ATL-P342S astrocytes as compared to those in WT astrocytes (Fig. 6d). Conversely, the number of LDs was increased in ATL1-P342S astrocytes (Fig. 6e), similar to previous results reported in *Atl1* mutated intestinal cells in *C. elegans* [30]. Similar changes in the size and number of LDs were observed in astrocytes with the ATL1-A161P mutation (Fig. 6f and g), revealing consistent LD defects in SPG3A astrocytes.

Next, we examined whether GW3965 can rescue the LD defects in SPG3A glial cells. GW3965 was able to suppress the changes in LDs (Fig. 6h–l), increasing the size of LDs (Fig. 6i and k) and reducing the number of LDs (Fig. 6j and l). Analysis of gene expression data revealed that the mRNA expression of the LD protein perilipin 2 (PLIN2), which was significantly reduced in SPG3A astroglial cells, was increased by GW3965 (Fig. 6m). PLIN2 is a LD protein that is universally expressed and regulates *SREBF1*, a gene critical for lipid and cholesterol metabolism [37]. Notably, the expression of* SREBF1* was significantly reduced in ATL1-P342S and ATL-A161P astrocytes (Fig. 6a and b), but not in neurons (Additional file 2: Fig. S6b and S6c); this reduction was mitigated by treatment with GW3965 (Fig. 6m). Taken together, these data reveal that *ATL1* mutations impair the size of LDs in SPG3A astrocytes, which can be rescued with GW3965 by, at least partially, regulating expression of genes important for LDs and lipid biogenesis.

### Increasing astrocyte-to-neuron cholesterol trafficking mitigates axonal defects

**Fig. 7:**
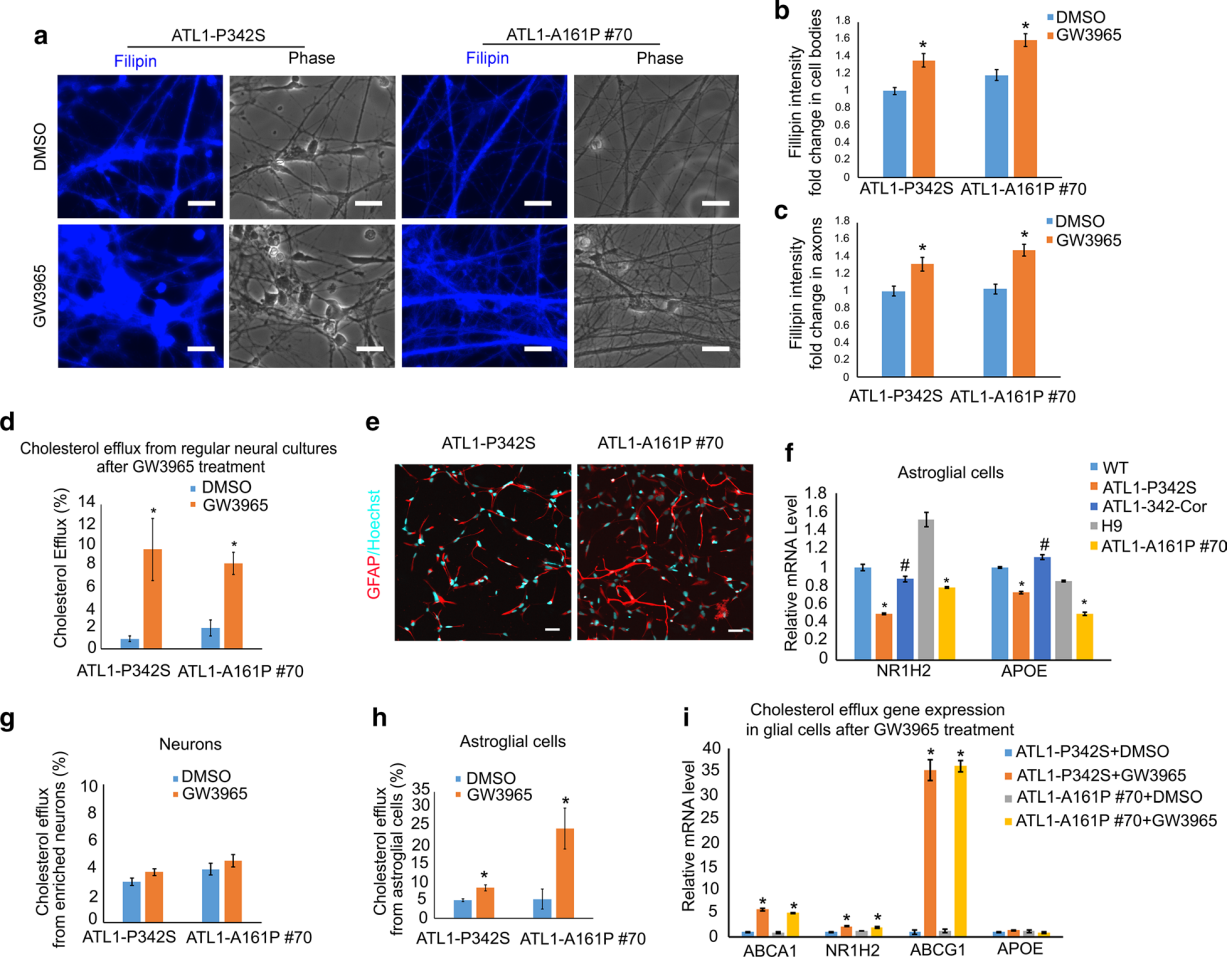
GW3965 treatment increases cholesterol content of SPG3A cortical PNs by promoting cholesterol efflux from astroglial cells in mutant ATL1 neural cultures. **a** Representative fluorescence and corresponding phase images of Filipin staining of ATL1-P342S and ATL1-A161P #70 cortical PNs after GW3965 or DMSO treatment for 3 days at 10 weeks in culture. Scale bar: 20 µm. **b**, **c** Quantification of Filipin staining intensity in cell bodies (**b**) and axons (**c**) of GW3965-treated ATL1-P342S and ATL1-A161P #70 cortical PNs. **d** Cholesterol efflux in ATL1-P342S and ATL1-A161P #70 regular neural cultures after GW3965 or DMSO treatment for 3 days. **e** Immunostaining of astroglial cell makers, GFAP in ATL1-P342S and ATL1-A161P #70 astroglial cells. Red: GFAP, cyan: Hoechst. Scale bar: 50 µm. **f** *NR1H2* and *APOE* mRNA levels in WT, ATL1-P342S, ATL1-342-Cor, H9 and ATL1-A161P #70 astroglial cells. Data are represented as mean ± SEM. *p < 0.05 compared to WT (for ATL1-P342S) and H9 (for ATL1-A161P #70). ^#^*p* < 0.05 compared to ATL1-P342S. **g** Cholesterol efflux from ATL1-P342S and ATL1-A161P #70 enriched cortical PNs at D49 after 1 µM GW3965 or DMSO treatment for 3 days. **h** Cholesterol efflux from ATL1-P342S and ATL1-A161P #70 astroglial cells after 1 µM GW3965 or DMSO treatment for 3 days. **i** Cholesterol efflux-associated gene expression in ATL1-P342S and ATL1-A161P #70 astroglial cells after GW3965 (1 µM) or DMSO treatment for 3 days. Data are represented as mean ± SEM. *p < 0.05 compared to DMSO treated ATL1-P342S and ATL1-A161P #70 astroglial cells, respectively, by two-sided Student’s *t*-test

To determine how GW3965 rescues axonal defects of cortical PNs, we first evaluated the cholesterol content in GW3965 treated ATL1-P342S and ATL-A161P #70 cortical PNs (10-week) by Filipin staining (Fig. 7a, b and c). The average Filipin intensity in GW3965-treated ATL1-P342S cortical PNs was significantly increased in both cell bodies and axons compared to those in DMSO-treated neurons, respectively (Fig. 7b and c). Since GW3965 activates NR1H2, which induces the reverse transport of cholesterol (cholesterol efflux), we then examined the cholesterol transfer in SPG3A cultures using a cholesterol efflux kit. Cholesterol efflux from both ATL1-P342S and ATL1-A161P #70 neural cultures into the medium was significantly increased by GW3965 as compared to vehicle (Fig. 7d). Together, these data reveal that treatment of GW3965 increases cholesterol efflux in human iPSC-derived neural cultures and restores cholesterol levels in cortical PNs with SPG3A mutant atlastin-1.

What is the source of cholesterol? Since liver X receptor agonists may not have any effect on the efflux of cholesterol from neurons [73], and since our cultures contain both neurons and astrocytes, we reasoned that astrocytes are likely the main source of cholesterol. To test this hypothesis, we generated enriched astroglial [31] and neuronal cells and examined the effects of GW3965 in these cultures separately. Immunostaining for GFAP showed a high proportion of astroglial cells in the glial culture (~ 95%, Fig. 7e). The enriched cortical PNs were obtained after incubation with compound E (Additional file 2: Fig.S7), a γ-secretase inhibitor that can enhance neuronal differentiation [5]. The mRNA expression levels of *NR1H2* and *ApoE*, critical regulators of cholesterol efflux, were significantly reduced in astrocytes (Fig. 7f), but not neurons (Additional file 2: Fig. S8a), with *ATL1* mutations. The ApoE secreted from SPG3A astrocytes was significantly reduced compared to WT astrocytes (Additional file 2: Fig. S8b). Next, we examined the efflux of cholesterol in the enriched neuronal and astroglial cultures after treatment with GW3965 (1 µM was selected given that enriched cultures were more sensitive to the drug treatment) [11]. Notably, GW3965 treatment significantly enhanced cholesterol efflux from both ATL1-P342S and ATL1-A161P #70 enriched astrocytes, but not cortical PNs, when compared to DMSO-treated groups (Fig. 7g and h). Moreover, after GW3965 treatment in enriched glial cells (Fig. 7i), mRNA expression of *ABCA1* and *ABCG1*, two downstream effectors of NR1H2, were significantly increased. ABCA1 and ABCG1 proteins are enriched in astrocytes and are important transporters for cholesterol efflux in glial cells [8]. Expression of these factors was also significantly increased by GW3965 in regular neural cultures that comprise both neurons and astrocytes (Additional file 2: Fig. S8c), revealing that GW3965 restores cholesterol levels in cortical PNs by regulating ABCA1/ABCG1-mediated cholesterol efflux from astrocytes. Taken together, these data demonstrate that impaired cholesterol transfer from glial cells to neurons underlies the cholesterol deficiency in SPG3A cortical PNs and serves as a potential therapeutic target for rescuing axonal degeneration in HSP.

## Discussion

HSP patients present with length-dependent axonal degeneration selectively in cortical motor neurons. Indeed, transgenic *Drosophila* and zebrafish models replicate axonal defects in motor neurons [17, 34], although these models do not distinguish cortical versus spinal motor neurons. In this study, using SPG3A patient iPSCs as a model system, we show reduced axonal length, impaired axonal transport, and accumulated axonal swellings in cortical PNs but not spinal motor neurons, recapitulating the hallmark HSP pathology. Importantly, we revealed that *ATL1* mutations dysregulate the expression of LD genes and alter the size of LDs in glial cells specifically, contributing to aberrant glia-neuron interactions and subsequently reduced levels of cholesterol in cortical PNs. Restoration of cholesterol homeostasis by correcting the LD defects in glial cells and increasing cholesterol efflux from glial cells was able to rescue axonal defects of cortical PNs. We have thus uncovered a non-cell autonomous mechanism contributing to cortical motor neuron degeneration in HSP.

Cholesterol is highly enriched in the brain, accounting for 25% of the total cholesterol in the body [57, 72]. As a major component of the plasma membrane, cholesterol is critical for maintaining normal axonal functions [41, 72]. Since the uptake of lipoprotein cholesterol is prevented by the blood brain barrier, cholesterol in the brain primarily comes from de novo synthesis [13, 56, 74]. Glial cells are the major source of cholesterol and provide cholesterol to neurons through lipoprotein-mediated transfer. Though neurons can synthesize cholesterol during development, this process gradually declines after birth and becomes totally dependent on glial cells [48]. It has been hypothesized that impaired transfer of lipid from glial cells to neurons may underlie neurological diseases, yet direct evidence is lacking. Using the SPG3A iPSC model and unbiased RNA-sequencing, we found that the axonal defects (transport and swellings) are accompanied by reduced cholesterol levels in neurons, and these pathological phenotypes become more prominent over time as astrocytes appear in the cultures. This phenomenon led us to hypothesize that the neuronal defects may be non-cell autonomous. Indeed, the simple application of cholesterol to the culture medium corrects most of the phenotypes, highlighting reduced production of cholesterol from HSP astrocytes as a cause. This is supported by the fact that the addition of conditioned media from normal astrocytes can rescue the axonal deficits. Thus, impaired lipid metabolism in glia appears responsible, at least partially, for the HSP-related axonal defects and provides a potential therapeutic target for HSP as well as other diseases involving axonopathy, such as amyotrophic lateral sclerosis and Alzheimer disease [23, 28, 42, 43].

Impaired axonal transport and subsequent accumulation of transported cargos are a pathological hallmark of HSP [54, 55]. Neurons are highly polarized, and transported cargos can move in both anterograde and retrograde directions. Reduced axonal transport of mitochondria has been observed in models of several HSP subtypes, including SPG3A [77]. Using SPG3A iPSC-based models, we also observed impaired axonal transport of synaptophysin, further supporting the implications of axonal transport in HSP. Interestingly, axonal transport in the anterograde direction is significantly reduced in SPG3A neurons as compared with control neurons. Further examination of genes for the anterograde and retrograde transport machinery revealed a significant reduction in the mRNA expression of *KIF1A*, *KIF3A*, and *KIF5A* in SPG3A cortical PNs (Additional file 2: Fig. S9). This is consistent with a study reporting reduced mRNA expression of kinesin-related genes, as well as impairment of anterograde synaptophysin transport, in SPG11 human neurons and spatacsin-silenced mouse neurons [55]. Moreover, one autosomal dominant HSP subtype, SPG30, is caused by mutation in *KIF1A*, which is involved in the anterograde transport of vesicles along axons [54]. Though these data support defective transport in the anterograde direction in HSP pathogenesis, impaired axonal transport in the retrograde direction has also been reported in some HSP models, including cortical neurons derived from iPSCs of SPG4, the most common form of HSP [12]. Whether anterograde and retrograde axonal transport are differentially affected in different HSP subtypes at different time points during disease progression, and how mutations of HSP genes affect different motor proteins, await further investigation.

Atlastin-1 can directly regulate LDs in intestinal cells [30] in vivo, but how atlastin-1 mutations lead to impaired lipid metabolism in SPG3A brain is unknown. It has been reported that the transmembrane regions of *ATL1* could potentially interact with cholesterol [33]. *ATL1* is important for cargo mobility which affects lipid trafficking and is mediated by membrane tethering [49, 53]. We found that atlastin-1 mutations alter the expression of genes involved in biogenesis and transfer of lipids, including *PLIN2*, *NR1H2*, and *SREBP1*. SREBP1 is the master regulator of lipid biogenesis [68], whereas PLIN2 participates in the fusion of LDs [37]. NR1H2 is a sterol sensor, regulating cholesterol efflux from glial cells to neurons [1]. Reduced expression of these genes in HSP cells suggests impaired lipid biosynthesis and/or homeostasis. Indeed, we found that LDs are enriched in astrocytes (with very few in neurons) and that their sizes are specifically reduced in SPG3A astrocytes. Smaller LDs coincide with decreased expression of NR1H2, a sterol sensor, leading to reduced cholesterol efflux from glial cells to neurons. The effect of mutant atlastin-1 on lipid homeostasis is supported by the pharmacological experiments with GW3965, which significantly increases the expression of *PLIN2*, *SREBP1* and *NR1H2* in SPG3A astrocytes. PLIN2 regulates lipid biogenesis and the fusion of LDs. The levels of SREBP1 are reduced in PLIN2-null cells [37]. Correspondingly, the size and number of LDs in HSP astrocytes are largely restored by GW3965 treatment. Furthermore, GW3965 treatment also increases the expression of *NR1H2*, a critical player in cholesterol efflux. This is accompanied by increased levels of cholesterol in the culture media. These findings suggest that the SPG3A mutations affect multiple steps in lipid metabolism in astrocytes, from biogenesis to LD formation and exocytosis, contributing to reduced cholesterol that impairs neuronal function. The crucial role of glial cells is further supported by the rescue of disease phenotypes using conditioned medium from normal astrocytes. By purifying neurons and glial cells and then co-culturing neurons with glial cells (including different types of glial cells), future studies will further dissect their roles and interactions in the pathogenesis of HSP.

We propose a non-cell autonomous model for cortical motor neuron degeneration in SPG3A. *ATL1* mutations impair lipid biogenesis and LD formation in glial cells and disrupt cholesterol transfer from glial cells to cortical PNs, resulting in axonal degeneration of SPG3A cortical PNs. Regulation of cholesterol homeostasis through glia-neuron interaction represents a novel pathway underlying axonal and neuronal degeneration in HSP. Though the detailed mechanisms by which *ATL1* mutations impair LDs and cholesterol trafficking await further investigation, our data point to a new target to treat axonal defects in HSP through regulating glial cells to restore cholesterol homeostasis.

## Supplementary information

**Additional file 1.** Supplementary Table 1. List of qRT-PCR primers.

**Additional file 2.** Supplementary Figures 1 to 9.

**Additional file 3.** Supplementary Table 2. List of Specifically Decreased GO Terms in SPG3A Cortical PN Cultures.
