# Supplementary material for: Impaired lipid metabolism in astrocytes underlies degeneration of cortical projection neurons in hereditary spastic paraplegia
Source: Acta Neuropathol Commun. 2020 Dec 7;8:214. doi: 10.1186/s40478-020-01088-0 (PMC7720406; doi:10.1186/s40478-020-01088-0)
Supplement: Supplementary file 1 — Additional file 1. Supplementary Table 1. List of qRT-PCR primers. [file 40478_2020_1088_MOESM1_ESM.docx]

| Gene | Forward Primer | Reverse Primer |
| --- | --- | --- |
| LDLR | GAGGTGGCCAGCAATAGAA | GATGACGGTGTCATAGGAAGAG |
| PLTP | CCAGCTGTCCAGCATGACTA | CGACTCCAGTGCAGAATGGT |
| LRP1 | CTGCTCTCAGCTCTGGTCG | CCAGCCCTTTGAGATACAGG |
| ABCA2 | AGCTGCTGCTCTGGAAGAAC | ATAAAGAACAGCACCAGGGG |
| ABCA1 | CATGAAGGACATGCGCAAAG | GTGATACAGGAACCCAGAGAAG |
| ABCG1 | CCTCTTCTTCTCCATGCTGTT | GTACCAGTAGTTCAGGTGTTCC |
| APOE | GATGAAGGTTCTGTGGGCTG | GCTCTGCCACTCGGTCTG |
| ABCG4 | CTCAAGTGCCTCTCAGGTAAAT | CTTCATTCCAGACTCCCTGTATC |
| NR1H2 | CGCTACAACCACGAGACAGA | ACTCGAAGATGGGGTTGATG |
| PLIN2 | ATGGCAGAGAACGGTGTGAAG | CAACTGCAATTTGCGGCTC |
| PLIN3 | TATGCCTCCACCAAGGAGAG | ATTCGCTGGCTGATGCAATCT |
| SREBF1 | GGGACAAGGAATTCTCGGATG | GAACTGATGGAGAAGCTGTAGG |
| KIF1A | GTCCGCCCCTTCAATTCCC | GAGGTGTGCGACCAGTAGG |
| KIF3A | GTGTTCGAGCTATTCCTGAACTT | CCTCTAACCTTTGTGTCTGATCC |
| KIF5A | ATGTTTTTGACCGTGTATTCCCC | TGAGGGTCGTGCAGCTTTC |
| DYNC1H1 | TTGGGCACTAGGAAATTGATGC | GCAGGGTTGATACGCCACA |
| DYNC1L12 | GGCTAGTGTTTTACGTGAGCA | TGGGGAACCTTGACAACCTTC |

**Supplementary Table 1. List of qRT-PCR primers**
