# Supplementary material for: Impaired lipid metabolism in astrocytes underlies degeneration of cortical projection neurons in hereditary spastic paraplegia
Source: Acta Neuropathol Commun. 2020 Dec 7;8:214. doi: 10.1186/s40478-020-01088-0 (PMC7720406; doi:10.1186/s40478-020-01088-0)
Supplement: Supplementary file 2 — Additional file 2. Supplementary Figures 1 to 9. [file 40478_2020_1088_MOESM2_ESM.pdf]

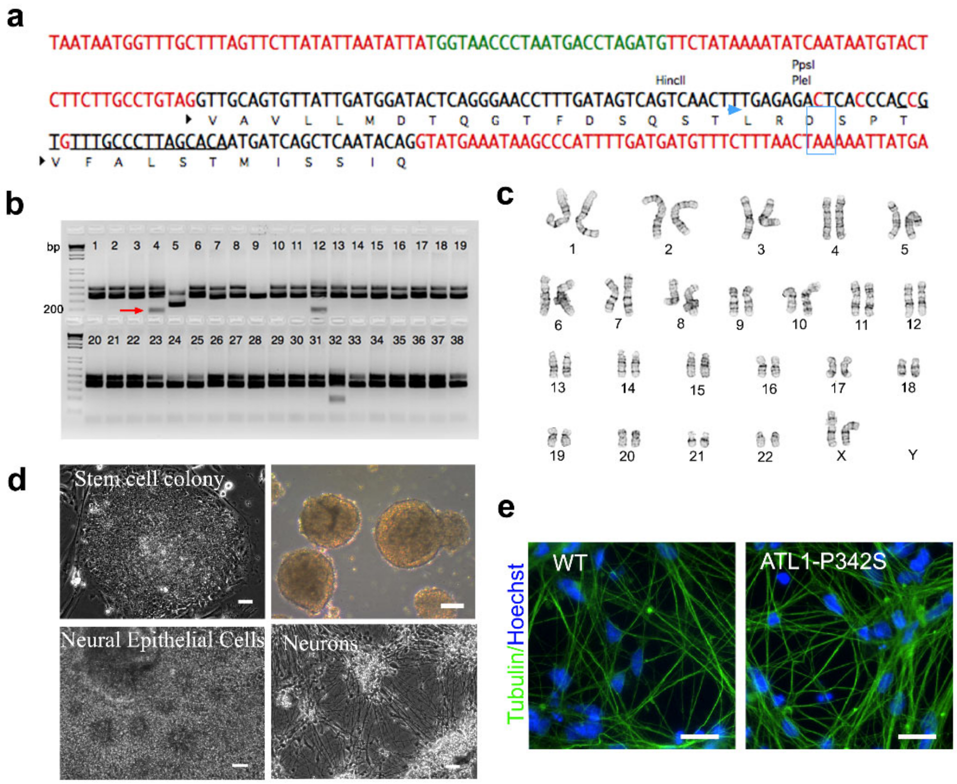

Supplementary Figure 1. Generation and differentiation of iPSCs with *ATL1* mutation. (**a, b**) Generation of isogenic cell lines in H9 hESCs (p.A161P in the ATL1 gene) using CRISPR-Cas9 homologous recombination. (**a**) Schematic strategy for knocking in the *ATL1* mutation (p.A161P, boxed area). A silent mutation creates a PleI site (arrowhead). (**b**) 200 bp band shows incorporation of ssODN template with PleI site in hESC clones. (**c**) The ATL1-A161P hESC line maintained a normal 46, XX female karyotype after 10 passages as shown by G-banded analysis. (**d**) Representative phase images of human pluripotent stem cells (hPSCs) at different stages during cortical projection neuron (PN) differentiation including stem cell colony, embryonic bodies (EBs), neuroepithelial cells, and cortical PNs. Scale bar: 50 μm. (**e**) Immunostaining images showing Tubulin^{+} neurites of WT and ATL1-P342S cortical PNs. Green: Tubulin, blue: Hoechst. Scale bar: 20 μm.

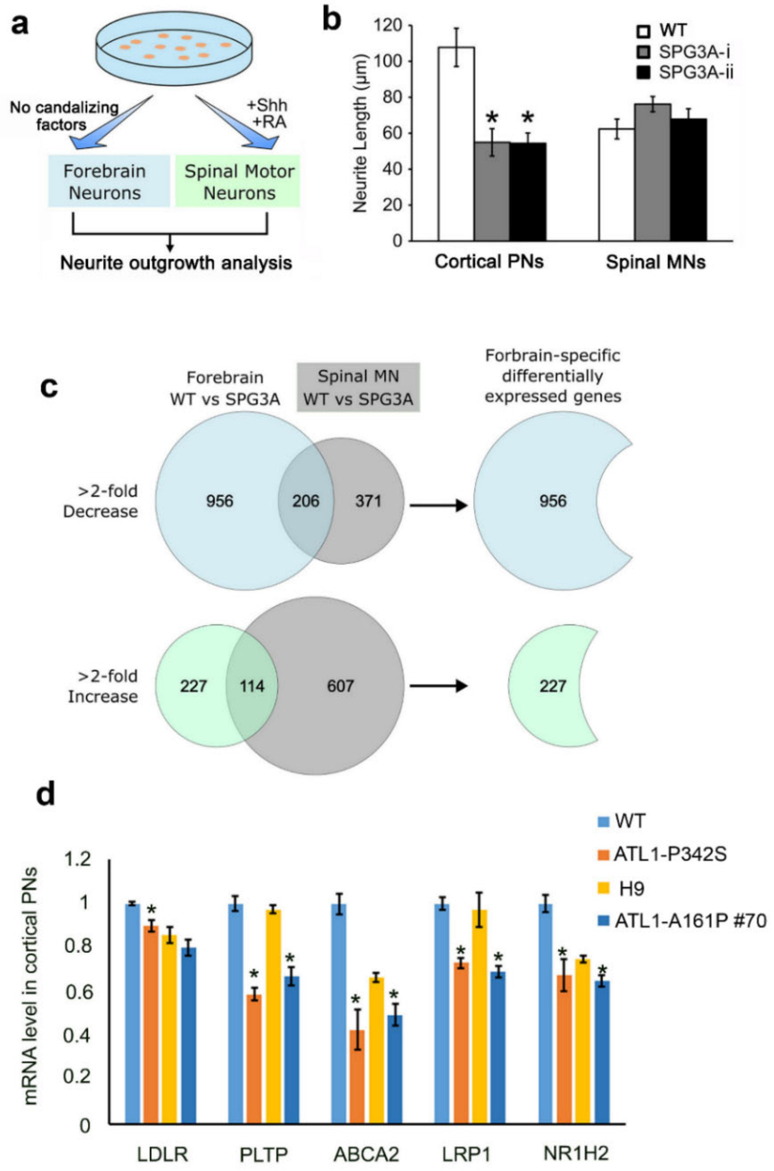

Supplementary Figure 2. mRNA-sequencing of cortical and spinal motor neurons to identify specific changes in SPG3A cortical PNs. (**a**) Schematic diagram of neural differentiation of cortical PNs and spinal motor neurons. (**b**) Analysis of axonal outgrowth of WT and SPG3A cortical PNs and spinal motor neurons. Axonal length of cortical PNs, but not spinal MNs, in SPG3A group was significantly reduced as compared to that in WT control group. *p < 0.05 compared to WT by ANOVA. (**c**) Venn diagrams depicting the number of genes differentially expressed between groups, either two-fold decreased or increased. Genes that were differentially expressed in spinal MN and forebrain cells were excluded from the forebrain list, to yield forebrain-specific differentially-expressed genes. (**d**) Lipid metabolism-associated gene expression in WT, ATL1-P342S, H9, and ATL1-A161P #70 cortical PNs. Data are represented as means ± SEM. *p < 0.05 compared to WT (for ATL1-P342S) and H9 (for ATL1-A161P #70) by two-sided Student's *t*-test.

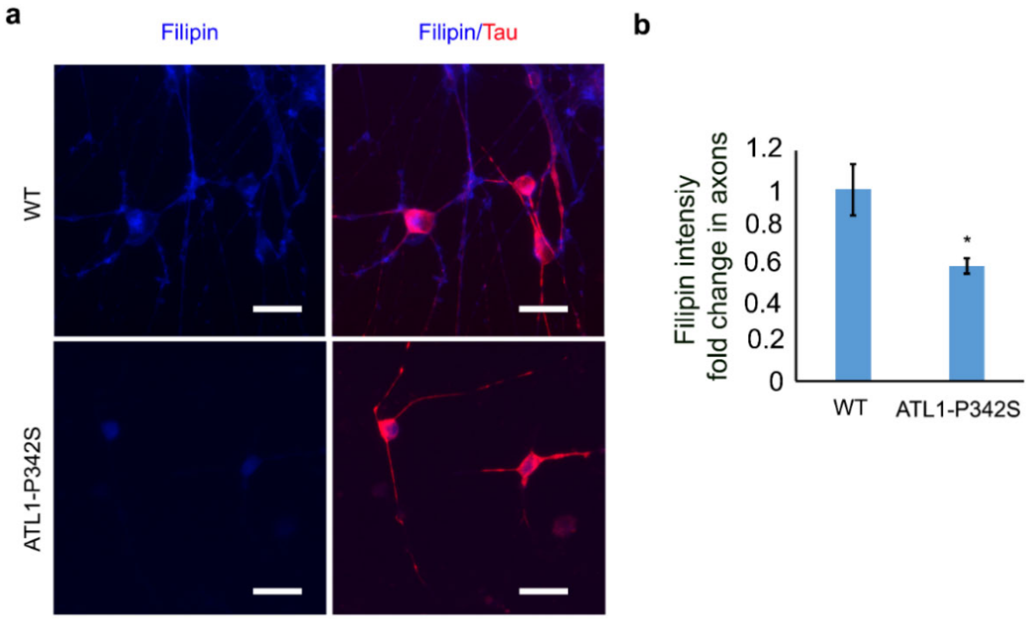

Supplementary Figure 3. Double staining of filipin with axonal marker, Tau in WT and ATL1-P342S cortical PNs. (**a**) Representative images of filipin with Tau in WT and ATL1-P342S cortical PNs. Blue: filipin. Red: Tau. Scale bar: 20 μm. (**b**) Quantification of filipin intensity in Tau positive axons of WT and ATL1-P342S cortical PNs. Data are represented as means ± SEM. * $p < 0.05$ compared to WT by two-sided Student's *t*-test.

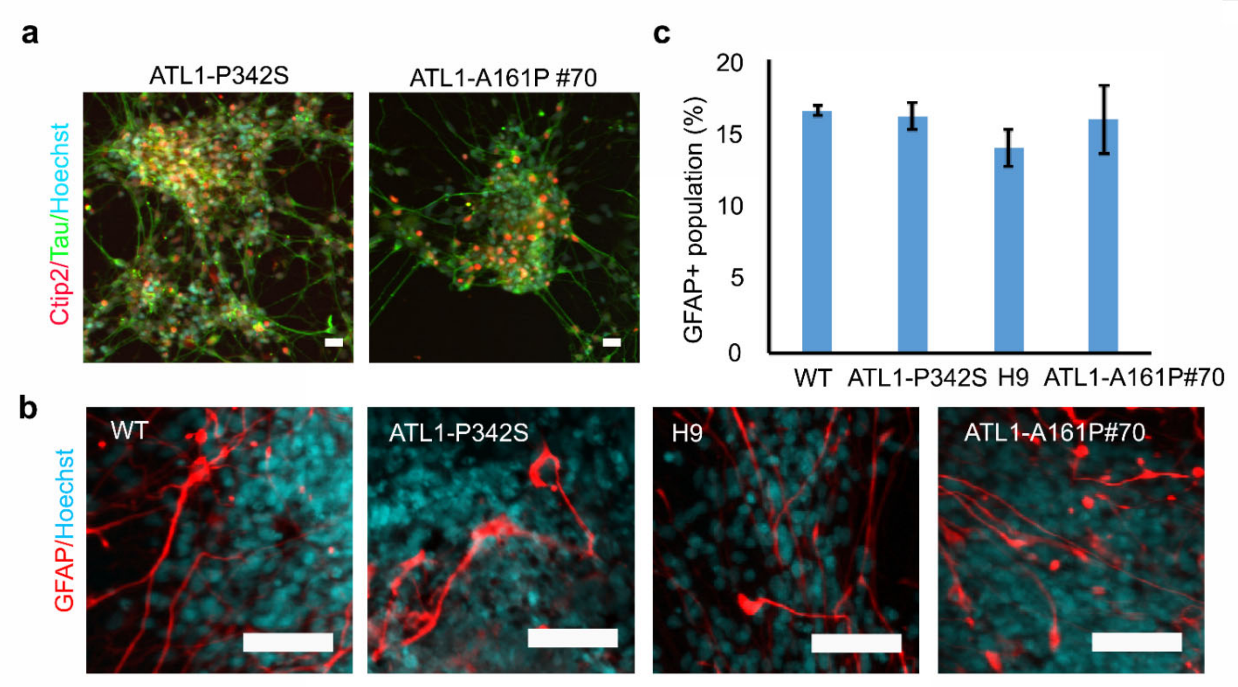

Supplementary Figure 4. Generation of cortical PNs and astrocytes in ATL1-P342S and ATL1-A161P regular neural cultures. (**a**) Representative images of Ctip2⁺ cortical PNs in ATL1-P342S and ATL1-A161P #70 regular neural cultures. Red: Ctip2; Green: Tau; Cyan: Hoechst. Scale bar: 20 µm. (**b**) Representative images of GFAP⁺ astrocytes in WT, ATL1-P342S, H9, and ATL1-A161P #70 regular neural cultures at 9 weeks. Red: GFAP; Cyan: Hoechst. Scale bar: 50 µm. (**c**) Quantification of GFAP⁺ cell population in WT, ATL1-P342S, H9, and ATL1-A161P #70 regular neural cultures at 9 weeks. Data are represented as means ± SEM.

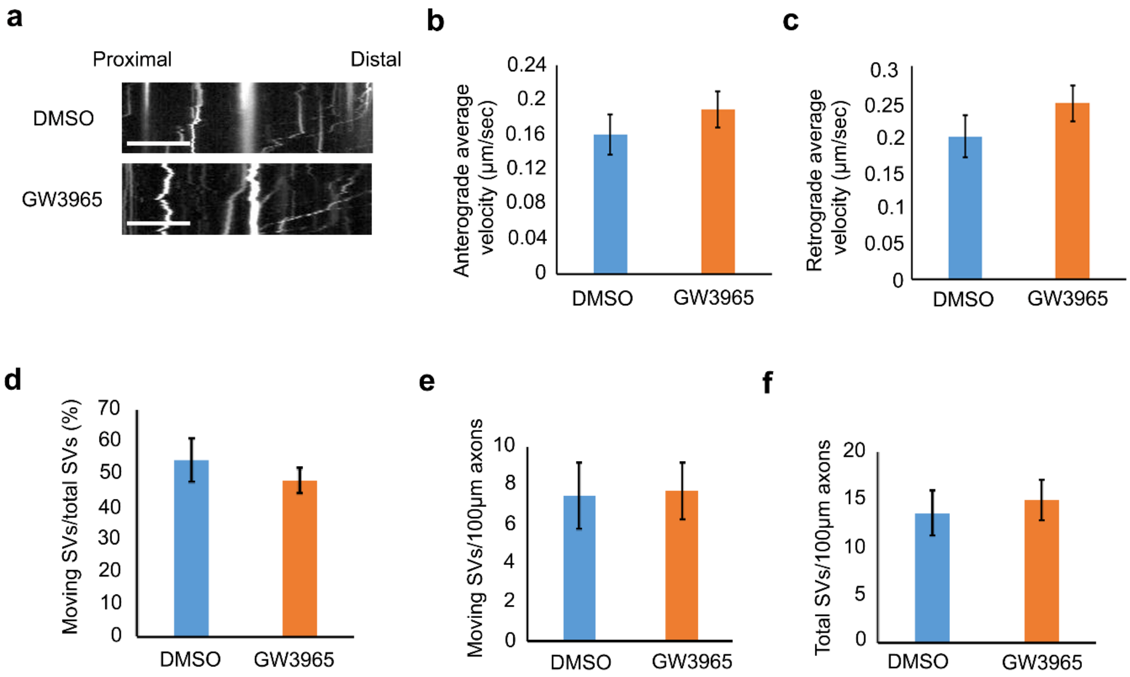

Supplementary Figure 5. The effects of GW3965 on synaptophysin transport of WT neurons. (**a**) Representative kymograph of synaptophysin transport in WT cortical PNs after treatment with GW3965 or DMSO for 3 days. Scale bar: 20 µm. (**b, c**) Anterograde (**b**) and retrograde (**c**) average moving velocities of SVs after GW3965 treatment for 3 days. (**d**) Ratio of moving SVs in relations to the total number of SVs after GW3965 treatment. (**e**) Ratio of moving SVs per 100 µm of axon after GW3965 treatment. (**f**) Number of total SVs per 100 µm of axon in WT cortical PNs after GW3965 treatment. Data are represented as means ± SEM.

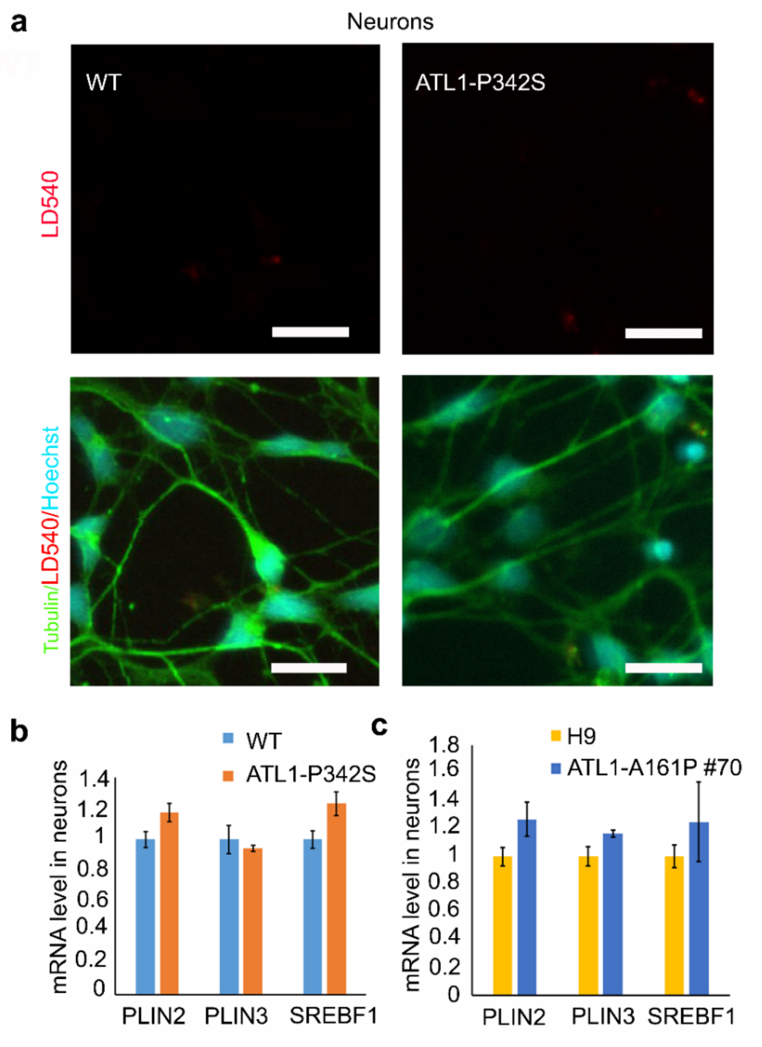

Supplementary Figure 6. LD and LD-associated gene expression in WT and ATL1-P342S neurons. (**a**) Visualization of LDs in WT and ATL1-P342S cortical PNs. Red: LD540; Green: tubulin; Cyan: Hoechst. Scale bar: 20 µm. (**b, c**) *PLIN2*, *PLIN3*, and *SREBF1* gene expression in WT, ATL1-P342S, H9 and ATL1-A161P #70 cortical PNs regular cultures. Data are represented as means ± SD.

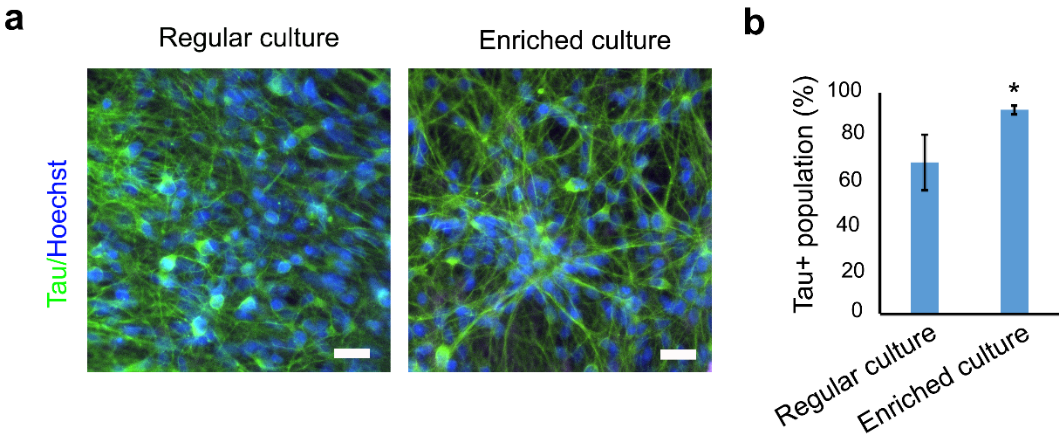

Supplementary figure 7. Tau⁺ neurons proportion in regular culture and enriched neural culture at 8 weeks. (**a**) Representative pictures of Tau⁺ neurons in regular culture and enriched neural culture. Green: Tau, blue: Hoechst. Scale bar: 20 μm. (**b**) Quantification of Tau⁺ neurons in regular culture and enriched neural culture at 8 weeks. Data are represented as means ± SD. *p < 0.05 compared to regular culture by two-sided Student's *t*-test.

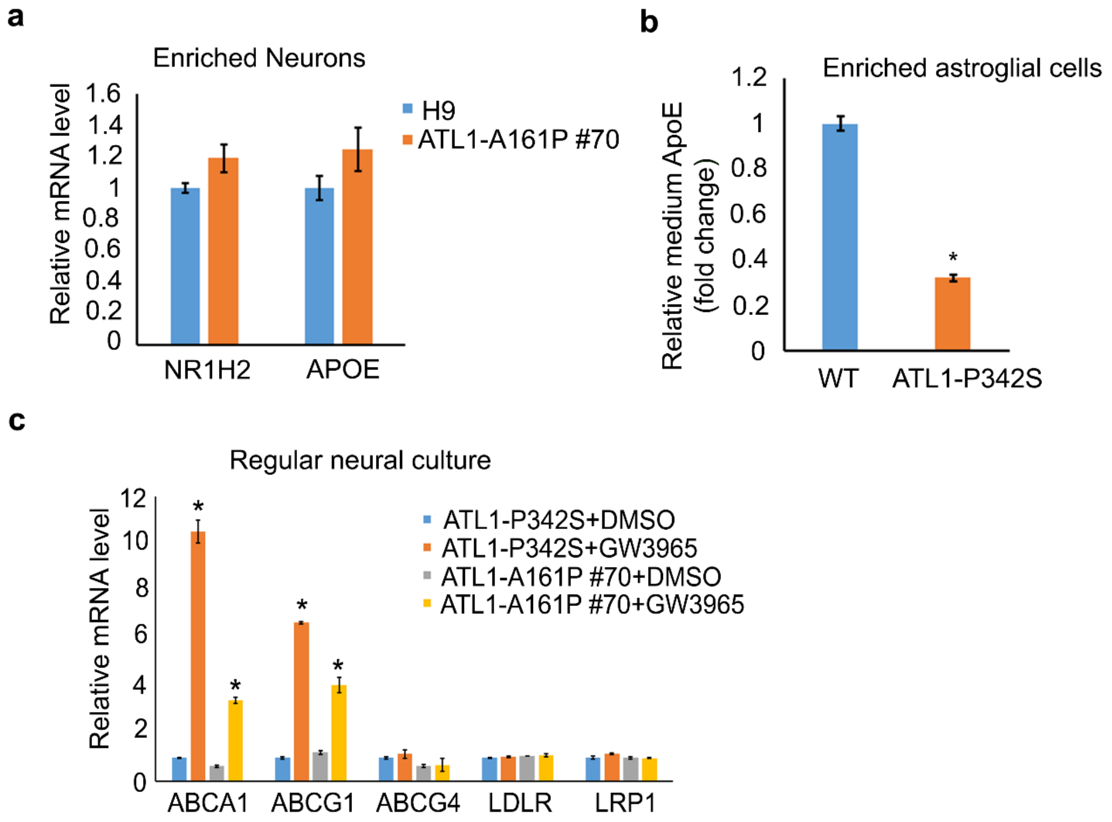

Supplementary Figure 8. Expression of cholesterol trafficking genes in cultures and the ApoE content in medium from enriched astroglial cells. (**a**) qPCR showing the expression of *NR1H2* and *ApoE* in enriched neurons. No significant changes were observed between control and *ATL1* mutant cortical PNs. (**b**) Relative ApoE content in culture medium of enriched astroglial cells derived from WT and ATL1-P342S cells. Data are represented as means ± SEM. *p < 0.05 compared to WT by two-sided Student's *t*-test. (**c**) *ABCA1*, *ABCG1*, *ABCG4*, *LDLR* and *LRP1* mRNA levels in ATL1 P342S and ATL161#70 regular neural cultures after 5 μM GW3965 treatment for 3 days. Data are represented as means ± SEM. *p < 0.05 compared to DMSO control by two-sided Student's *t*-test.

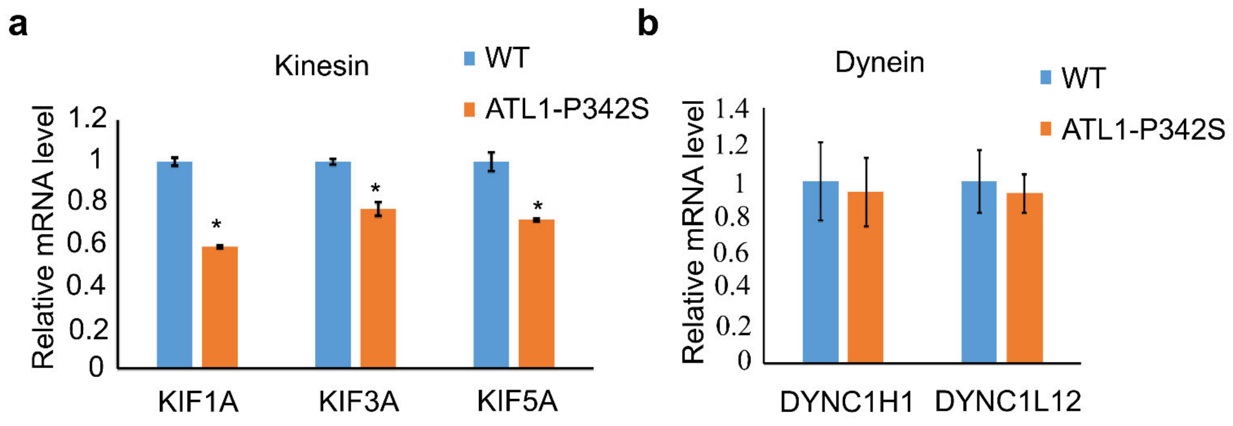

Supplementary figure 9. The mRNA expression of Kinesin and Dynein-related genes in WT and ATL1-P342S cortical PNs. (**a**) qPCR showing the mRNA expression of kinesin-related genes, *KIF1A*, *KIF3A*, and *KIF5A*. (**b**) mRNA expression of dynein-related genes, *DYNC1H1* and *DYNCL12*. Data are represented as means ± SD. * $p < 0.05$ compared to WT by two-sided Student's *t*-test.
