# Supplementary material for: Impaired lipid metabolism in astrocytes underlies degeneration of cortical projection neurons in hereditary spastic paraplegia
Source: Acta Neuropathol Commun. 2020 Dec 7;8:214. doi: 10.1186/s40478-020-01088-0 (PMC7720406; doi:10.1186/s40478-020-01088-0)
Supplement: Supplementary file 3 — Additional file 3. Supplementary Table 2. List of Specifically Decreased GO Terms in SPG3A Cortical PN Cultures. [file 40478_2020_1088_MOESM3_ESM.pdf]

Supplementary Table 2. List of Specifically Decreased GO Terms in SPG3A Cortical PN Cultures

GOTerm_BP_DIRECT	Count	%	PValue	Genes	List Total	Pop Hits	PopTotal	Fold Enrichment	Bonferroni	Benjamini	FDR
GO:0098609~cell-cell adhesion	35	3.6998	2.31E-07	ALDOA, ABCF3, SNX5, GIPC1, TAGLN2, CDC42EP1, PAK4, FASN, RAB11B, FAM129B, RPL7A, EHD1, HSPA8, PLEC, TES, UNC45A, DBNL, BSG, TWF2, CKAP5, TBC1D10A, FSCN1, LYPLA2, FLNB, EIF4G1, EIF4G2, CORO1B, SCYL1, STXBP6, TMOD3, NOP56, EEF1D, DBN1, PUF60, SH3GL1	799	271	16792	2.714278457	7.60E-04	7.60E-04	4.20E-04
GO:0060070~canonical Wnt signaling pathway	15	1.5856	3.34E-05	DVL2, DVL3, TCF7, SMAD3, FZD3, PTPRU, FZD2, SNAI2, WNT2B, SMO, CHD8, SDC1, PYGO2, BCL9L, MYC	799	83	16792	3.798121145	0.10392477	0.0533875	0.0606891
GO:0030335~positive regulation of cell migration	22	2.3256	1.81E-04	COL18A1, HRAS, ACTN4, MYO1C, BCAR1, CSF1, F2RL1, SMAD3, SNAI2, MCAM, MYADM, DAPK3, CORO1A, SEMA6B, ZNF703, ITGAV, SEMA3F, HBEGF, LAMC2, CTSH, PLAU, CIB1	799	184	16792	2.512814932	0.44866141	0.18001458	0.3288607
GO:0042157~lipoprotein metabolic process	9	0.9514	3.45E-04	APOL2, SDC1, BMP1, LRP1, APOE, ZDHHC8, LCAT, HSPG2, AMN	799	193	16792	4.977537712	0.67818324	0.24681413	0.6252857
GO:0006364~rRNA processing	23	2.4313	5.63E-04	TBL3, NOC4L, TSR1, RPL13, EXOSC4, RPLP2, RPL36, BOP1, DIS3L, RPS8, RPS3, SENP3, WDR18, CDKN2A, RPL18A, DHX37, RPL13A, NOL11, NOP58, NPM3, NOP56, RPL7A, WDR43	799	214	16792	2.258758027	0.84304544	0.30951415	1.0192596
GO:0008285~negative regulation of cell proliferation	35	3.6998	6.50E-04	DLC1, LZTS2, HRAS, TFAP4, HIST1H2AE, FGFRL1, IGFBP6, PML, ADORA1, SRF, MSX2, BAK1, CDKN2A, BCL6, QSOX1, TES, CIB1, COL18A1, TESC, NACC2, AIMP2, SIRT6, WNK2, PTPRU, SLC9A3R1, MXD4, TMEM115, HDAC4, CDKN1A, ADM, IRF1, HGS, RBM38, EMP3, BMP7	799	396	16792	1.857498641	0.88210983	0.29976105	1.1758606
GO:0050900~leukocyte migration	16	1.6913	6.72E-04	ICAM1, HRAS, BSG, F2RL1, SLC7A8, ITGA3, SLC7A5, GAS6, SLC7A6, MIF, SLC16A3, PROCR, ITGAV, SHC1, MSN, PROS1	799	122	16792	2.756232175	0.89023193	0.2706682	1.2148809
GO:0007160~cell-matrix adhesion	13	1.3742	1.11E-03	CTGF, ITGAV, ITGA7, ITGA11, ITGB5, BCAM, ITGA3, ADAMTS12, ZYX, EDA, SRF, PXN, ADAM15	799	90	16792	3.035683493	0.97375821	0.36558392	1.9938708
GO:0033235~positive regulation of protein sumoylation	5	0.5285	1.28E-03	HDAC4, CDKN2A, PIAS4, TOLLIP, GNL3	799	11	16792	9.552850154	0.98504517	0.37310045	2.2982914
GO:0008283~cell proliferation	32	3.3827	1.38E-03	FGF5, HRAS, PPARD, TSPO, APC2, CSF1, BCAR1, SIPA1, POLA1, BOP1, MIF, BAK1, BOK, PAK4, MYC, ERCC2, GNL3, ARHGEF1, FSCN1, PIM1, BRAT1, FURIN, GAS6, DDIT4, IGSF8, LRP1, SERPINF1, PPP1R8, LAMA5, FPGS, BIN1, EMP1	799	366	16792	1.837488117	0.98930187	0.3647703	2.479181
GO:0032469~endoplasmic reticulum calcium ion homeostasis	5	0.5285	4.54E-03	BAK1, WFS1, GRINA, BAX, PML	799	15	16792	7.005423446	0.99999968	0.52658747	7.9410715
GO:0010875~positive regulation of cholesterol efflux	4	0.4228	0.026283	NR1H2, LRP1, APOE, PLTP	799	14	16792	6.004648668	1	0.82648118	38.398969
